# A Double-Blind, Placebo-Controlled, Randomized, Clinical Trial of the TLR-3 Agonist Rintatolimod in Severe Cases of Chronic Fatigue Syndrome

**DOI:** 10.1371/journal.pone.0031334

**Published:** 2012-03-14

**Authors:** David R. Strayer, William A. Carter, Bruce C. Stouch, Staci R. Stevens, Lucinda Bateman, Paul J. Cimoch, Charles W. Lapp, Daniel L. Peterson, the Chronic Fatigue Syndrome AMP-516 Study Group, William M. Mitchell

**Affiliations:** 1 Hemispherx Biopharma, Inc., Philadelphia, Pennsylvania, United States of America; 2 BCS Consulting, Philadelphia, Pennsylvania, United States of America; 3 University of the Pacific, Stockton, California, United States of America; 4 Fatigue Consultation Clinic, Salt Lake City, Utah, United States of America; 5 Center for Special Immunology, Fountain Valley, California, United States of America; 6 Hunter-Hopkins Center, Charlotte, North Carolina, United States of America; 7 Sierra Internal Medicine Associates, Incline Village, Nevada, United States of America; 8 Vanderbilt University School of Medicine, Nashville, Tennessee, United States of America; Mayo Clinic, United States of America

## Abstract

**Background:**

Chronic fatigue syndrome/myalgic encephalomyelitis (CFS/ME) is a severely debilitating disease of unknown pathogenesis consisting of a variety of symptoms including severe fatigue. The objective of the study was to examine the efficacy and safety of a TLR-3 agonist, rintatolimod (Poly I: C_12_U), in patients with debilitating CFS/ME.

**Methods and Findings:**

A Phase III prospective, double-blind, randomized, placebo-controlled trial comparing twice weekly IV rintatolimod versus placebo was conducted in 234 subjects with long-standing, debilitating CFS/ME at 12 sites. The primary endpoint was the intra-patient change from baseline at Week 40 in exercise tolerance (ET). Secondary endpoints included concomitant drug usage, the Karnofsky Performance Score (KPS), Activities of Daily Living (ADL), and Vitality Score (SF 36). Subjects receiving rintatolimod for 40 weeks improved intra-patient placebo-adjusted ET 21.3% (p = 0.047) from baseline in an intention-to-treat analysis. Correction for subjects with reduced dosing compliance increased placebo-adjusted ET improvement to 28% (p = 0.022). The improvement observed represents approximately twice the minimum considered medically significant by regulatory agencies. The rintatolimod cohort vs. placebo also reduced dependence on drugs commonly used by patients in an attempt to alleviate the symptoms of CFS/ME (p = 0.048). Placebo subjects crossed-over to receive rintatolimod demonstrated an intra-patient improvement in ET performance at 24 weeks of 39% (p = 0.04). Rintatolimod at 400 mg twice weekly was generally well-tolerated.

**Conclusions/Significance:**

Rintatolimod produced objective improvement in ET and a reduction in CFS/ME related concomitant medication usage as well as other secondary outcomes.

**Trial Registration:**

ClinicalTrials.gov NCT00215800

## Introduction

Chronic fatigue syndrome/myalgic encephalomyelitis (CFS/ME) is a debilitating disorder characterized by disabling fatigue and a combination of flu-like symptoms [1]–[4]. The fatigue is not improved by bed rest and may be worsened by physical activity. The Centers for Disease Control and Prevention (CDC) has identified CFS/ME as an economically and emotionally devastating illness whose functional impairment can be equivalent to multiple sclerosis, heart disease, chronic obstructive pulmonary disease, or end-stage renal disease [4]. The etiologic basis for CFS/ME is unknown and may be multifactorial with a variety of viruses and immunological abnormalities linked to its pathogenesis [5]–[8] that may be familial [7] and dependent on recently discovered genetic signatures [9], [10].

CFS/ME is estimated to occur in at least 1 million individuals in the US, occurs most often in persons 40 to 59 years of age and is seen 3 times more frequently in women. Productivity among people with CFS/ME is estimated to decline by 37% in the household and by 54% in the labor force [11]. The resulting annual economic loss is of a significant magnitude estimated at $20,000 for each individual. With at least 1 million persons affected, the US national loss from CFS/ME greatly exceeds that estimated for other illnesses such as infectious and parasitic diseases ($10 billion), digestive system illnesses ($8.4 billion), and nervous system disorders ($6.4 billion) [11]. These productivity estimates do not include the significant health care costs for persons with CFS/ME, nor do they address the negative impact on the individual and family that results from the severe debilitation and reductions in quality of life.

With no approved drug therapy available, treatment is aimed at symptom relief and improved ambulatory function [12]. These include over the counter and off-label prescription drugs, behavioral modifications, and graded exercise therapies. The rationale for the initial trials with rintatolimod (Poly I:C_12_U) in CFS/ME were based on its recognized antiviral and immunomodulatory properties and as an inducer of interferon [13]. These properties are mediated by its activity as a selective toll-like receptor 3 (TLR-3) agonist in the induction of innate immune responses [14]. Toll-like receptors, as primordial transmembrane, pattern recognition receptors, trigger alarm signals against invading pathogens by modulating cytokine cascades. The initial success of open-label trials provided the basis for the double-blind, placebo controlled Phase II [15] and the current Phase III clinical trial as well as its FDA designation as an Orphan Drug for CFS and an FDA authorized treatment protocol for use of rintatolimod in CFS.

Cardiopulmonary exercise tolerance (ET) testing is an objective measurement of treatment efficacy for fatigue and is accepted as a regulatory standard for drugs ameliorating exertional fatigue by exhibiting an average improvement of at least a 6.5% in intra-group, placebo-adjusted ET [16]–[19]. The Phase III multi-center, double-blind, placebo controlled trial reported here uses ET as its primary endpoint and the reduction in drug usage for symptom relief as one of several secondary endpoints in the evaluation of rintatolimod in the treatment of CFS/ME.

## Methods

### Trial design

This study was a prospective, double-blind, Phase III trial with equal parallel cohorts conducted at 12 centers in the U.S. to evaluate the safety and efficacy of rintatolimod in CFS. The protocol for this trial and supporting CONSORT checklist are available as supporting information; see Checklist S1 and Protocol S1. The design of the study, including endpoint, and the statistical method used to define efficacy were all reviewed by the FDA prior to receipt of FDA authorization for the initiation of the study. The objective of this study was to compare in a blinded, randomized, placebo-controlled trial, changes in exercise tolerance, concomitant medication usage and other quality of life endpoints including safety parameters in patients with CFS/ME treated with rintatolimod vs. placebo. The inclusion and exclusion criteria for study enrollment is detailed in Table S1 and meets both the original [1] and revised [2] CDC clinical definitions of CFS. All patients gave written informed consent and had a diagnosis of CFS ≥12 months resulting in significant debilitation (KPS score of 40–60). A list of Principal Investigators in the CFS AMP-516 Study Group is shown in Table S2 along with the 12 study sites and Institutional Review Boards. This study was initiated on 12/17/98, and ended following full enrollment on 8/16/04 when the last patient completed.

Patients were stratified according to their treadmill duration (≤9 minutes vs. >9 minutes) and randomized to receive either rintatolimod (n = 117) or placebo (n = 117). Following a 200 mg IV dose or equal volume of placebo (physiological saline) twice weekly for two weeks, 400 mg was administered IV twice weekly versus placebo IV to 234 randomized patients with severe CFS for a total of 40 weeks (Stage 1). At the conclusion of Stage 1, the blind was continued and patients randomized to placebo were crossed-over to rintatolimod, while the original rintatolimod arm of the study continued on rintatolimod for 24 weeks (Stage 2). The primary endpoint was ET on a treadmill (Trackmaster TM 225, Full Vision, Inc., Newton, KS) with ECG monitoring performed by a team of exercise physiology specialists who traveled to each of the trial sites (Workwell Physiology Services, Inc., Ripon, CA). Secondary endpoints included changes in the use of concomitant medications to treat CFS/ME symptoms, Karnofsky Performance Score (KPS) (Table S3), Activities of Daily Living (ADL), and the Vitality and General Health Perceptions subscales from the Short Form 36-Health Survey (SF-36).

### Quality assurance of clinical and laboratory data and randomization

Rintatolimod (Ampligen®) used in this study was manufactured in accordance with Good Manufacturing Practice (GMP) and tested under Good Laboratory Practice (GLP) and Good Clinical Practice (GCP) guidelines. Prior to the initiation of the study, vials of active and placebo drug were prepared and labeled with a unique allocation number. After providing informed consent and meeting the entrance criteria, patients were first stratified by duration of ET, and then randomized and assigned to receive an allocation number that tied back to the blinded drug supply provided for the study. At the site and patient level, only the allocation number was known, which provided no insight to the randomization assignment of the study drug. The patient randomization schedules utilized a permuted block design size of 4 and were provided by an independent group, Simirex, Inc., Mt. Laurel, NJ. Knowledge of randomization schedules and patient assignments was restricted to the individuals responsible for preparing and packaging the study medication; none of these individuals had any other operational role in the study. Unblinding of the study occurred following final field audits for data accuracy and database lock.

### Exercise tolerance (ET) testing

Patients underwent treadmill ET testing according to a standardized protocol (Table S4). Subjects rated their perceived exertion, generally considered to be a reliable indicator of fatigue [20], using the Borg Scale and progressed through stages successively until they could no longer continue. The ET result was recorded as the total time on the treadmill. In order to reduce variation in test results, each site used the same make and model of treadmill (Trackmaster TM 225, Full Vision, Inc., Newton, Kansas USA) and the same group of exercise physiologists traveled to each site to administer the treadmill test throughout the study. Treadmills were calibrated on the day of each test for speed and inclination. Two treadmill exercise tests were performed during baseline. If the two baseline tests differed by more than ±10% of the maximum duration from their mean value, a third test was performed. When three baseline tests were performed, the two closest tests were used for data analysis of ET.

### Sample size

The sample size for this clinical investigation was based on detecting a difference in the intra-patient changes in ET (seconds) between the randomized treatment groups using a 2-tailed test and a type 1 error rate of 5%. With a minimum of 100 patients enrolled per treatment arm, a mean difference between the active treatment arm and placebo arm equivalent to 40% of the common standard deviation would have 80% power to detect a difference using a 2-tailed analysis with an alpha of 5%.

### Statistical analysis

Data analyses used SAS (Version 8.2 and 9.1) statistical software. All statistical analyses were two-sided. The primary endpoint (intra-patient changes in treadmill duration, week 40 minus baseline) was analyzed using a one-factor (treatment assignment) analysis of covariance test (ANCOVA) with the mean of two baseline treadmill ET serving as the covariate. Although the design of the study considered repeated measurements on the patients over time, the statistical model for evaluating efficacy was predicted on a landmark analysis based on the intra-patient changes at week 40. The 2-sample t-test was used to compare baseline ET between the two randomized treatment groups; the intra-group changes (also known as “within group changes”) in ET from baseline to week 40 were analyzed using a paired-difference t-test. The proportion of patients who achieved a 25% to 50% increase in ET at week 40 (intra-patient changes or within subject changes) was compared between randomized treatment groups using a two-tailed chi-square test in a continuous responder analysis, calculated using 5% increments from ≥25% improvement to ≥50% improvement. Secondary endpoints were analyzed based on the distribution of the dependent variable. The normality of the distributions was examined using the Shapiro-Wilk test.

The intent-to-treat (ITT) population included all patients who received study drug and performed the ET study parameter at least once during the treatment phase. The last ET observation was used for patients who failed to complete the week 40 visit. A completer group, consisting of all patients who competed the 40 weeks of Stage 1, was also pre-specified. All analyses were conducted using the statistical software from the SAS Institute (Cary NC).

## Results

### Demographic and other baseline characteristics

**Figure 1:**
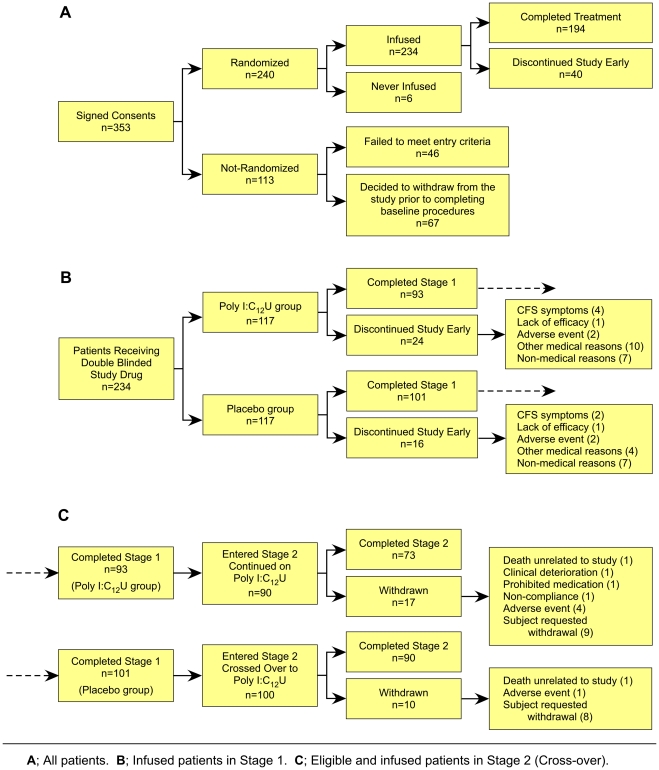
Flow Diagram of Study Patients.

Of the 234 patients with CFS/ME admitted to the study, an equal number (n = 117) were randomized to each study arm, rintatolimod versus placebo (Figure 1). The two study arms were well balanced. There was no statistically significant difference with regard to age (43.4 versus 43.5 years), duration of CFS symptoms (9.6 versus 9.7 years), time from diagnosis (5.9 versus 5.9 years), percentage female gender (67.5 versus 77.8), or body weight (167 versus 166 pounds) between the rintatolimod and placebo cohorts, respectively (Table S5).

### Primary endpoint analysis

The primary efficacy endpoint of the study was improvement in treadmill ET at week 40. One hundred ninety four patients (194) completed all 40 weeks of Stage 1. In addition, seven rintatolimod and seven placebo patients, who discontinued the study, completed a treadmill ET test following initial dosing and were included in the ITT analysis. The reasons for discontinuation are presented in Figure 1. An analysis of the primary endpoint, treadmill ET, at 40 weeks in the ITT group is shown in Table 1A. By week 40, the rintatolimod cohort (n = 100) had a mean change increase in ET of 96 seconds to 672, corresponding to a group mean increase of 16.6% and an intra-patient mean increase of 36.5%. In contrast, the placebo group (n = 108) increased ET by 28 seconds to 616 corresponding to an intra-group mean increase of 4.8% and an intra-patient mean increase of 15.2%. The placebo-adjusted intra-group and intra-patient increases in the rintatolimod ITT cohort were 11.8% and 21.3%, respectively (p = 0.047). For the patients who completed all 40 weeks (Table 1B) of the study (n = 194), mean baseline ET was 583 seconds for the rintatolimod cohort (n = 93) compared to 587 seconds for the placebo group (n = 101). At week 40, the rintatolimod patients had increased mean ET by 108 seconds (18.6%) to 691 compared to an increase of 27 seconds (4.6%) to 614 in the placebo cohort. The placebo-adjusted, intra-group and intra-patient increases were 14.0% and 24.6%, respectively.

**Table 1:** Analysis of the Effect of Rintatolimod on the Primary Endpoint, Exercise Tolerance (ET).

A. Increase in Exercise Treadmill Duration with Rintatolimod in CFS Patients (Intent-to-Treat)					
Study Interval	Mean (SD) Exercise Duration (Seconds)		Percent Increase from Baseline^1^		p-value
	Rintatolimod (n = 100)	Placebo (n = 108)	Rintatolimod (n = 100)	Placebo (n = 108)	
Baseline	576 (257.5)	588 (234.4)	-	-	0.729^2^
Week 40	672 (314.1)	616 (286.7)	36.5	15.2	0.047^3^
p-value^4^	-	-	<0.001	0.198	

B. Increase in Exercise Treadmill Duration with Rintatolimod in CFS Patients (Completer Population)					
Study Interval	Mean (SD) Exercise Duration (Seconds)		Percent Increase from Baseline^1^		p-value
	Rintatolimod (n = 93)	Placebo (n = 101)	Rintatolimod (n = 93)	Placebo (n = 101)	
Baseline	583 (254.7)	587 (237.3)	-	-	0.908^2^
Week 40	691 (311.4)	614 (291.2)	40.2	15.6	0.019^3^
p-value^4^	-	-	<0.001	0.244	

C. Increase in Exercise Treadmill Duration with Rintatolimod in CFS Patients without Significant Dose Reductions (Intent-to-Treat)					
Study Interval	Mean (SD) Exercise Duration (Seconds)		Percent Increase from Baseline^1^		p-value
	Rintatolimod (n = 83)	Placebo (n = 98)	Rintatolimod (n = 83)	Placebo (n = 98)	
Baseline	581 (256.2)	590 (235.3)	-	-	0.813^2^
Week 40	690 (308.2)	616 (291.4)	43.0	15.0	0.022^3^
p-value^4^	-	-	<0.001	0.263	

D. Frequency Distribution of Percent Change from Mean Baseline Exercise Treadmill Duration at Week 40 (Intent-to-Treat)			
Improvement from Mean Baseline Exercise Treadmill Duration	Rintatolimod (n = 100)	Placebo (n = 108)	p-value^5^
At least 25%, n (%)	39 (39)	25(23)	0.013
At least 50%, n (%)	26 (26)	15 (14)	0.028

E. Effect of Baseline ET on Week 40 ET (Intent-to-Treat)								
	Mean (SD) Exercise Duration Mean (seconds)				% Gain Rintatolimod over Placebo^1^		p-value^3^	
Baseline ET Strata (Minutes)	≤9 Drug (n = 40)	≤9 Placebo (n = 42)	>9 Drug (n = 60)	>9 Placebo (n = 66)	≤9	>9	≤9	>9
Baseline	321 (153.3)	353 (144.6)	747 (148.3)	738 (137.7)				
Week 40	450 (284.2)	446 (264.6)	820 (237.3)	725 (245.9)	31.0	15.0	0.517	0.034

1 Mean intra-patient percent improvement.

2 Student's t-test comparing mean baseline ET between treatment groups.

3 Analysis of covariance (ANCOVA) with baseline as a covariate comparing the mean ET change from baseline within each treatment group.

4 Paired t-test comparing whether the change from baseline is equal to zero within each treatment group.

5 Probability that a difference between treatment groups exists using the chi-square test.

The intra-patient change in ET relates to the individual patient responses. The statistic is utilized to determine the proportion of rintatolimod versus placebo patients that increased ET by ≥25% and ≥50%. The placebo-adjusted mean intra-treatment group change of 11.8% is important for showing efficacy. A pre-defined minimum of 6.5% increase in mean intra-treatment group ET was required in the protocol to show efficacy. Table S8 provides a comparison of the intra-group ET benefit achieved by rintatolimod compared to current medical and regulatory standards of care for cardiotropic drugs providing benefit for exertional fatigue symptoms.

At 40 weeks, the difference in improvement in ET for the rintatolimod versus placebo cohorts in the pre-specified completer and ITT groups was statistically significant (p = 0.019 and 0.047, respectively) using an analysis of covariance model. A paired-difference t-test for analysis of the intra-patient difference from baseline provided additional evidence that rintatolimod produced a significant increase in ET for patients debilitated with CFS/ME. Both the completer and ITT populations improved ET significantly (p<0.001) compared to the placebo cohorts (p≥0.198).

The effect of a pre-specified dose modification analysis was performed by exclusion of patients in the ITT population with significant dose reductions, defined as a combined total of 20 missed doses or dose reductions of at least 50%. Table 1C demonstrates that when patients with significant dose reductions were excluded, the placebo-adjusted intra-patient mean improvement in ET was 28% (p = 0.022) with ET in the rintatolimod arm significantly enhanced (p<0.001) from baseline compared to the placebo arm (0.263).

Additional evidence supporting the efficacy of rintatolimod in CFS/ME was provided by an ad hoc analysis of the frequency distribution of percent improvement in ET from baseline to week 40 in the rintatolimod versus placebo cohorts (Table 1D). The proportions of patients in the ITT population with increases in ET from baseline to week 40 of at least 25% and of at least 50% were 1.7 and 1.9-fold greater for patients randomized to rintatolimod than placebo, 39% versus 23% (p = 0.013) and 26% versus 14%, respectively (p<0.028). A continuous responder analysis, calculated using 5% increments from ≥25% improvement to ≥50% improvement revealed a significant difference at 40 weeks in favor of the patients who received rintatolimod compared tp placebo. The proportion of patients with decreases in ET from baseline to week 40 of at least 25% were not statistically significantly different between the rintatolimod versus placebo cohorts, 17% versus 19%, respectively (p = 0.76).

Although separate analyses of the two baseline ET stratification subgroups was not powered adequately, the results demonstrated a medically significant increase in ET in both subgroups. Table 1E demonstrates that medically significant, placebo-adjusted enhancement of treadmill ET of 15% and 31% was seen in the patients stratified to the high and low baseline performance cohorts, respectively. Stage 2 results support the conclusions of Stage 1. The Stage I double-blinded status was maintained in Stage 2. The original placebo patients crossed-over to receive rintatolimod achieved a mean intra-patient percent improvement in ET of 39% (p = 0.040) in 24 weeks, while the original rintatolimod cohort maintained their improvement in ET (Table 2).

**Table 2:** Mean/Median Baseline and Mean/Median Change from Baseline in Exercise Treadmill Duration (seconds) at Week 24 (Stage 2) (ITT Population).

	Treatment Cohorts			
	Prior Double-Blinded Randomized Treatment (Stage 1)↓Double-Blinded Cross-over Treatment (Stage 2)			
Exercise Tolerance (ET) Test Parameter	Rintatolimod (Stage 1)↓Rintatolimod (Stage 2)(n = 72)		Placebo (Stage 1)↓Rintatolimod (Stage 2)(n = 90)	
	Mean (SD)	Median	Mean (SD)	Median
Baseline ET (seconds)^1^	706 (308)	726	626 (291)	638
ET at End of Stage 2 (seconds)	696 (323)	732	669 (288)	665
ET Difference (seconds)	−10.4 (160.36)	14.0	43.1 (198.26)	46.0
Percent ET Improvement^2^	22.9	1.7	39.0	8.9
p-value^3^	0.58	0.69	0.04	0.02

1 Baseline of Stage 2 is the last non-missing Stage 1 value (last observation carried forward [LOCF]).

2 Mean intra-patient percent improvement.

3 p-value for the mean is from a paired t-test; p-value for the median is from the Wilcoxon signed rank test.

### Secondary endpoint analysis

A variety of secondary endpoints were studied for evidence of efficacy. These endpoints were discussed and agreed upon with the FDA prior to the initiation of the study. Concomitant medications were recorded on the case report forms (CRF) with a determination as to whether the concomitant medication was taken to alleviate CFS/ME-related symptoms. Subjects were neither counseled or encouraged to discontinue medications before starting study drug. Table 3 illustrates the change in concomitant drug usage for the relief of CFS symptoms at the conclusion of Stage 1. Rintatolimod treatment was associated with a significant reduction in drug usage at 40 weeks as compared to placebo in both analyses of patients who completed 40 weeks of treatment (p = 0.010) and the ITT population (p = 0.015) taking CFS/ME-palliative drugs, and the total completer (p = 0.029) and ITT populations (p = 0.048). Table S9 shows the most frequently used drug categories and examples of each.

**Table 3:** Effect of Rintatolimod (PolyI:C_12_U) on the Use of Concomitant Medications for the Relief of CFS Symptoms (Stage I).

	Number (%) of Patients		
Direction of Change^1^	Rintatolimod	Placebo	p-value^2^
1. Change from initial medication use to end of study use (intent-to-treat)			
Decrease	68 (68) (n = 100)	59 (55) (n = 108)	0.048
No decrease	32 (32) (n = 100)	49 (45) (n = 108)	0.048
2. Change from initial medication use to end of study by the cohort who used concomitant medications (intent-to-treat)			
Decrease	68 (72) (n = 94)	59 (56) (n = 106)	0.015
No decrease	26 (28) (n = 94)	47 (44) (n = 106)	0.015
3. Change from initial medication use to end of study use in patients completing 40 weeks			
Decrease	64 (69) (n = 93)	54 (53) (n = 101)	0.029
No decrease	29 (31) (n = 93)	47 (47) (n = 101)	0.029
4. Change from initial medication use to end of study by the cohort who used concomitant medications in patients completing 40 weeks			
Decrease	64 (73) (n = 88)	54 (55) (n = 99)	0.010
No decrease	24 (27) (n = 88)	45 (45) (n = 99)	0.010

1 Daily average of the number of concomitant medications used during the first 4 weeks of study compared to the daily average of the number used during the final 4 weeks of study.

2 Chi-square test.

Significant changes (p<0.01) from baseline in secondary performance endpoints were observed in KPS, ADL, and vitality scores for the patients receiving rintatolimod during Stage 1 (Table 4). Although the placebo cohort did not improve the median KPS score, the Wilcoxon signed rank analysis indicated a significant shift in individual scores. Similarly, a significant change was seen with regard to general health perceptions for the placebo cohort, but not for the rintatolimod cohort, despite having the same baseline and week 40 median scores. During Stage 2, additional support for the efficacy of rintatolimod in CFS/ME was seen (data not shown). Analysis of the ITT population showed that median KPS increased from 50 to 60 (p<0.001) and mean ADL improved from 71.3 to 72.4 (p = 0.04), no significant change in vitality or general health perceptions scores was seen during Stage 2.

**Table 4:** Secondary Performance Endpoint Improvements in Stage 1.

	Rintatolimod			Placebo		
Secondary Performance Endpoint	Baseline	Week 40	p-value^1^	Baseline	Week 40	p-value^1^
Karnofsky Performance Score (KPS)	50	55	<0.01	50	50	<0.01
Activities of Daily Living (ADL)	68.1	72.4	<0.01	68.7	69.4	ns^2^
Vitality Score (SF-36)	5.0	10.0	<0.01	10.0	10.0	ns^2^
General Health Perception (SF-36)	20	20	ns^2^	20	25	<0.01

1 p-value derived from a Wilcoxon signed rank test comparing whether the median change from Baseline is equal to 0 within each treatment group.

2 ns = not significant, p>0.05.

### Safety assessment

During Stage 1, 99% of rintatolimod patients reported at least one adverse event (AE) compared to 97% in the placebo cohort (Table 5). Four AEs (flu-like syndrome, chills, vasodilatation and dyspnea) were reported more frequently in the rintatolimod compared to the placebo cohort (p<0.05, Fisher's exact test). These symptoms were generally mild or moderate in severity. There was no statistically significant difference in the frequency of injection site reactions between the two study groups (see Table S6 for the frequency of adverse events). The frequency of serious AEs during Stage 1 was not significantly different between the two study arms. No serious AEs were definitely related to study drug. Table S7 summarizes the serious AEs during Stage 1. There were no clinically significant differences between the two treatment cohorts with regard to the number or percentage of patients with AEs in Stage 2. There were three serious AEs in each study arm judged by the site PI as not related (n = 5) or remotely related (n = 1) to the study drug. There were two deaths in Stage 2, neither in the opinion of the principal investigators at the clinical sites was related to study drug. A 59 year-old male died of respiratory failure related to pneumonia and lung disease, a pre-existing condition. The other death occurred in a 40 year-old male, who was shooting a firearm at motor vehicles and committed suicide when confronted by police. The investigator concluded that the death was related to pre-existing conditions of CFS/ME related depression and a history of violent behavior. Neither of these pre-existing conditions disqualified entry into the trial. Notably the three most prevalent causes of death in CFS/ME patients are heart failure, suicide and cancer [21], [22]. Also, these studies typically enroll subjects with less severe symptomatology than those treated in this clinical trial of rintatolimod.

**Table 5:** Safety- Adverse Events (AEs) of Rintatolimod (PolyI:C_12_U).

	Rintatolimod (n = 117)		Placebo (n = 117)	
A. Stage 1: AEs During 40 Weeks of Drug Exposure	Number of Patients	% of Patients	Number of Patients	% of Patients
Patients with ≥1 AE	116	99.1	113	96.6
All AEs with p≤0.05				
Flu-like syndrome	62	53.0	46	39.3
Chills	26	22.2	11	9.4
Vasodilatation	25	21.0	11	9.4
Dyspnea	14	12.0	4	3.4
Patients with ≥1 Serious AE	12	10	6	5

B. Stage 2: AEs During 24 Weeks Cross-over Study	Rintatolimod → Rintatolimod (n = 90)		Placebo → Rintatolimod (n = 100)	
Patients with ≥1 AE	86	95.6	98	98.0
Patients with ≥1 Serious AE	3	3.3	3	3.0

There were no clinically significant changes from baseline hematology, chemistry, or coagulation laboratory tests, all mean values remained within the normal range. No patient discontinued secondary to a grade 3 or 4 laboratory toxicity. There were no statistically significant differences in physical exam findings, or post-infusion vital signs.

## Discussion

CFS/ME patients exhibiting both the Holmes [1] and Fukuda [2] diagnostic criteria were selected for this study. The primary endpoint of efficacy, improvement in ET, achieved statistical significance by several analytical methods applied to the ITT cohort. The intra-patient placebo-adjusted enhancement in mean ET at Week 40 was 21.3% (p = 0.047). Correction for patients with significant missed doses or dose reductions improved mean intra-patient ET enhancement to 28% (p = 0.022). The proportions of patients in the ITT population with changes from mean baseline ET duration at week 40 of at least 25% and of at least 50% were 1.7 and 1.9-fold greater for subjects randomized to rintatolimod than placebo, 39% versus 23% (p = 0.016) and 26% versus 14% (p = 0.036), respectively. At week 40, the ITT population randomized to rintatolimod had a placebo-adjusted mean intra-treatment group change from baseline ET of 11.8%, 1.8-fold higher than pre-defined in the protocol as the minimum 6.5% required to show efficacy (16–19). Table S8 provides a comparison of the intra-group benefit achieved by rintatolimod to current medical and regulatory standards of care for cardiotropic drugs providing benefit for exertional fatigue symptoms.

There is no specific treatment for CFS/ME and patients utilize a large number of drugs in an attempt to alleviate the debilitating symptoms of the illness. Decreases in concomitant medication usage is an objective measure of the effect of rintatolimod on the symptoms of CFS/ME. Rintatolimod was associated with a statistically significant reduction in the use of concomitant medications used to help treatment symptoms to CFS/ME relative to placebo: 68% of patients receiving rintatolimod decreased use of concomitant medications related to CFS versus 55% of subjects receiving placebo (P = 0.048). Other secondary endpoints provided additional evidence of efficacy during Stage 1 and the blinded Stage 2 cross-over. The data from this Phase III trial, moreover, supports an earlier double-blinded, controlled Phase II trial [15], which also demonstrated a greater mean intra-patient ET improvement with rintatolimod compared to placebo (31% vs. 16% (p = 0.01)). The decrease in concomitant medication usage associated with rintatolimod treatment has been reported previously to decrease the prolonged cardiac QT intervals induced by many of the drugs used by patients with CFS/ME to alleviate the symptoms of CFS/ME [23].

Rintatolimod was generally well-tolerated in patients with CFS/ME. The total number of AEs were statistically equivalent between cohorts, although patients receiving rintatolimod had an increased incidence in four AEs (flu-like syndrome, chills, vasodilatation and dyspnea) of usually mild severity. Two deaths, one from each arm of Stage 2 were unrelated to rintatolimod treatment. No significant differences were observed between rintatolimod and placebo in mean hematological, or blood chemistry parameters.

A potential limitation of this trial is that only severely debilitated cases of CFS were enrolled. All patients had a diagnosis of CFS for at least 12 months and a baseline KPS score of 40–60. Therefore, the findings may not be relevant for patients with CFS disease of a lesser severity. A potential source of bias exists since a commercial entity, Hemispherx Biopharma, Inc. funded and conducted this trial using independent investigators. This concern is mitigated since this study remained blinded to all Hemispherx clinical trial staff involved with the study until data base lock and the clinical data and statistical analysis were reviewed and audited by the FDA. This audit included a comparison if the data utilized for the statistical analysis versus the data contained in source documents at the investigator sites.

Differential gene expression in peripheral blood of CFS/ME patients has been reported by a number of investigators using DNA microchip analysis [24]–[34]. Recently, rigorous patient selection and a microchip that surveys the entire human genome coupled with qPCR gene validation has provided a more complete appreciation of the gene expression profiles that occur in CSF [9]–[10]. A complex array of differential gene expression can be categorized into functional subsets relating to responses to infection, immunity, inflammation, apoptosis, neurological function, and cancer [10]. Many of these differential gene responses are consistent with the large number of infectious agents linked with CFS/ME as well as the altered immune responses and variety of signs and symptoms observed with the disease. For example, eIF4G1 is an eukaryotic mitochondrial translation factor utilized in replication by a variety of viruses including the enteroviruses implicated in the pathogenesis of CFS/ME [9], [35]. EIF4G1 variant 5 (GenBank:NM_004953) is upregulated in CFS/ME suggesting a physiological response to viral replication as well as a gene variant favoring pathogen persistence.

Prior to this report, no treatment had been demonstrated to be active in multiple randomized controlled clinical trials. The observed activity of rintatolimod in CFS/ME provides a rational intervention in this complex, multi-factorial disease. Rintatolimod is an activating ligand (dsRNA) for TLR3, which is a first line defense mechanism essential in the induction of innate immunity. Although the mechanism of action of rintatolimod in CFS is unknown, certain genes may be modulated by rintatolimod including those for metabolic enzymes, antiviral response elements, and immune mediators. Subsequent modulation of host defense responses may depend on transient expression of multiple genes and modulation of a number of enzymes regulated by TLR 3 activation. Among the hundreds of upregulated genes triggered by this process [36] are the interferons with broad anti-viral activities mediated by interferon's own upregulation of anti-viral genes including 2–5′ adenylate synthetase and protein kinase (p68) activated by rintatolimod [37]. The availability of diagnostic assays, based on gene expression profiles, may provide a prognostic genetic signature that will predict response, as well as the opportunity to develop synergistic combinations of rintatolimod and other agents to expand the therapeutic effect in this debilitating disease. The current study more clearly establishes the safety profile of rintatolimod. Other TLRs including those for TLR4 and TLR9 are under development for human use. Their safety, however, based on clinical trials has been questioned based on safety issues, pharmacokinetics, and biodistribution [38]. The safety profile of rintatolimod is likely related to the exclusive use of the TRIF intracellular signaling pathway [39] as well as the restriction of rintatolimod as a TLR3 agonist, a property not exhibited by the toxic TLR3 ligand, Poly I:C [19], [40]. The pathway of TLR3 induction of innate immunity is unique among the TLRs by its exclusive utilization of TRIF as the molecular mediator of NF-κB, IRF-3/IRF-7 transcription factors [39]. All the other TLRs utilize a MyD88/TiRAP adapter mediated pathway. The safety profile and unique innate immune stimulating properties of rintatolimod provides strong motivation to assess its potential role in microbial and cancer vaccines. Rintatolimod activates dendritic cells and potentiates both humoral and cell-mediated responses [40]. For example, Japanese investigators have demonstrated that rintatolimod has powerful adjuvant activity when coupled with various influenza vaccines including highly pathogenic avian influenza virus (HPAIV) [41]–[43]. The Japanese government has invested significant effort in providing preclinical data in support of clinical trials of rintatolimod for seasonal and H5N1 HPAIV vaccines.

## Supporting Information

Checklist S1 (DOC)

Protocol S1 (PDF)

Table S1 **Requirements for Entry into the Phase-III AMP-516 Clinical Study.**
(DOC)

Table S2 **Clinical Trial Sites, Principal Investigators, and IRB.**
(DOC)

Table S3 **Clinical Significance of Karnofsky Performance Scale Scores.**
(DOC)

Table S4 **Treadmill Exercise Testing Protocol.**
(DOC)

Table S5 **Demographic Characteristics of the Rintatolimod (Poly I:C_12_U) versus Placebo Cohorts.**
(DOC)

Table S6 **All Adverse Events Experienced by at Least 5% of Subjects in Either Treatment Group during the First 40 Weeks (Safety Population).**
(DOC)

Table S7 **Chronological Listing of Stage I Serious Adverse Events (SAEs).**
(DOC)

Table S8 **Comparison of Intra-Group Mean ET Improvement Seen With Rintatolimod (PolyI:C_12_U) vs. Approved Drugs for Non-CFS Severe Exertional Fatigue.**
(DOC)

Table S9 **Concomitant Medications Used for CFS Symptoms.**
(DOC)
