# Supplementary material for: A Double-Blind, Placebo-Controlled, Randomized, Clinical Trial of the TLR-3 Agonist Rintatolimod in Severe Cases of Chronic Fatigue Syndrome
Source: PLoS One. 2012 Mar 14;7(3):e31334. doi: 10.1371/journal.pone.0031334 (PMC3303772; doi:10.1371/journal.pone.0031334)
Supplement: Protocol S1 — (PDF) [file pone.0031334.s002.pdf]

CLINICAL PROTOCOL

A MULTI-CENTER, DOUBLE-BLIND, RANDOMIZED, PLACEBO-CONTROLLED STUDY OF THE EFFICACY AND SAFETY OF POLY I:POLY $C_{12}U$ (AMPLIGEN®) 400 MG IV TWICE WEEKLY VERSUS PLACEBO IN PATIENTS WITH SEVERELY DEBILITATING CHRONIC FATIGUE SYNDROME (CFS)/MYALGIC ENCEPHALOMYELITIS (ME)

PROTOCOL NO. AMP-516

Investigator Statement of Agreement:

I have carefully read this protocol and I agree to conduct this study as described.

Date ______ Principal Investigator ______

Date ______ Representative: Hemispherx Biopharma, Inc.

Sponsor:

Hemispherx Biopharma, Inc.
One Penn Center, Suite 660
1617 JFK Blvd.
Philadelphia, PA 19103
Phone: 215/988-0080
Fax: 215/988-1739

CONFIDENTIAL

Version 4/20/03

SCHEMA

Title: A multi-center, double-blind, randomized, placebo-controlled study of the safety and efficacy of poly I:poly $C_{12}U$ (Ampligen®) 400 mg IV twice weekly versus placebo in patients with severely debilitating chronic fatigue syndrome (CFS)/myalgic encephalomyelitis (ME).

Protocol No.: AMP-516

Design: Prospective, double-blind, randomized, placebo-controlled, equal parallel groups, multi-center.

Population: 230 -240 patients with CFS/ME at up to 15 study centers (approximately 10 to 30 patients per center).

Stratification: According to treadmill duration ($\leq$ 9 minutes vs. $>$ 9 minutes).

Regimen:

Baseline Evaluations

↓

STAGE I: 40 weeks, double-blind, randomized, placebo-controlled

Randomization into 1 of 2 parallel arms with 115 patients per arm:

1. Poly I:poly $C_{12}U$ 200 mg by intravenous infusions (IV) twice weekly for 4 doses (Weeks 1 and 2) then 400 mg IV twice weekly for 76 doses (Weeks 3 through 40);
2. Placebo IV twice weekly for 80 doses (Weeks 1 through 40).

To maintain the treatment blind, the volumes of poly I:poly $C_{12}U$ (Ampligen®) and placebo infused will be equal at any time during the study.

↓

STAGE II: 24 weeks, open-label evaluation

Patients who comply with the protocol requirements of STAGE I, whether they received poly I:poly $C_{12}U$ or placebo, are eligible to participate in a 24 week open-label study. Patients will receive poly I:poly $C_{12}U$ 200 mg IV twice weekly for 4 doses (Weeks 1 and 2) then 400 mg IV twice weekly for 44 doses (Weeks 3 through 24).

Primary Endpoint:
1. Treadmill Exercise Tolerance Testing (ETT): Duration

Secondary Endpoints:
1. Karnofsky Performance Score (KPS)
2. Activities of Daily Living (ADL)
3. Symptoms Check List-90-Revised (SCL-90-R); CFS/ME Dimension and Cognition
4. Activity Monitoring

5. Short Form-36, Health Survey
6. Concomitant Medications used to relieve symptoms of CFS/ME
7. Frequency of hospitalizations/emergency room/outpatient visits and procedures.

Efficacy will be established by showing medically and statistically significant improvement as defined in Section 11.0 Statistical Considerations of the primary endpoint, ETT.

Schematic of Study

Prohibited Medication Washout	Baseline	Randomized, Double-Blind	Open-Label	Follow-up
8 Weeks (Week -20 to -13)	12 Weeks (Week -12 to 0)	40 Weeks (Week 1-40)	24 Weeks (Week 41-64)	Two Years
		STAGE I	STAGE II	

TABLE OF CONTENTS

		Page
1.0	Introduction	1
2.0	Objectives	2
3.0	Study Design	2
3.1	Primary Endpoint	3
3.2	Secondary Endpoints	3
4.0	Patient Selection	3
4.1	Inclusion criteria	3
4.2	Exclusion Criteria	4
5.0	Concomitant/Prohibited Medications	6
5.1	Concomitant Medications	6
5.2	Prohibited Medications	6
6.0	Study Medication	7
6.1	Dosing Schedule	7
6.2	Dosage Modification	8
6.3	Drug Preparation	9
6.4	Drug Supplies	9
6.5	Blinding Procedures	9
7.0	Evaluations and Assessments	9
7.1	Evaluation Methods	9
7.2	Scheduling	14
8.0	Management of Intercurrent Events	17
8.1	Departure from Protocol	17
8.2	Adverse Events	17
8.3	Serious Adverse Events	18
9.0	Reasons for Discontinued Participation	19
10.0	Administrative Aspects	19
10.1	Institutional Review Board (IRB) and Informed Consent	19
10.2	Study Commencement and Discontinuation	19
10.3	Data Recording	20
10.4	Monitoring of Study	20
10.5	Drug Accountability	20
10.6	Investigator's Final Report	20
10.7	Disclosure of Data	21
11.0	Statistical Considerations	21
11.1	Endpoint	21
11.2	Sample Size	22
11.3	Randomization	22
11.4	Subset Analyses	23
11.5	Dropouts and Dose-Reductions	23

TABLE OF APPENDICES

		Page
Appendix A	Toxicity Criteria	24
Appendix B	Time and Event Schedule	25
Appendix C	Patient Consent Form	29
Appendix D	Case Definition of CFS	35
Appendix E	Procedure for Determining Karnofsky Performance Score (KPS)	38
Appendix F	Exercise Tolerance Testing (ETT) Procedure	41
Appendix G	Assessment of CFS/ME Signs and Symptoms	46
Appendix H	Locator Questionnaire	47

1.0 Introduction

The investigational drug poly I:poly $C_{12}U$ (Ampligen®) is a specific form of mismatched, double-stranded ribonucleic acid (dsRNA) in which uridylic acid (U) substitutions in the polycytidylic acid chain creates periodic regions of non-hydrogen bonding in the molecular configuration. Double-stranded RNAs act as modulators of lymphokines, molecules that mediate cellular immune and antiviral activities, including interferon, interleukin and tumor necrosis factor (TNF). Also, dsRNAs directly activate intracellular pathways associated with antiviral and immune enhanced states.

Myalgic encephalomyelitis (ME), also known as chronic fatigue syndrome (CFS), is a disorder characterized by chronic or recurrent debilitation associated with viral reactivation and immune abnormalities for which no effective treatment is known. The Center for Disease Control (CDC) has established strict case definitions of CFS to distinguish it from other diseases. An analysis of a randomized placebo-controlled study suggests that poly I:poly $C_{12}U$ (Ampligen®), a broad spectrum antiviral with immune modulator capacities, may be an effective treatment for a certain subset of CFS/ME patients, namely those with severe debilitation.

A multi-center, double-blind, randomized clinical trial of poly I:poly $C_{12}U$ (Ampligen®) vs. placebo was performed with 92 patients meeting the 1988 CDC case definition of CFS. Forty-five (45) patients entering the study with Karnofsky Performance Scores (KPS) between 20 and 60 were treated with poly I:poly $C_{12}U$ up to 24 weeks and showed median KPS improvement of over 8 points; 47 patients receiving placebo showed no improvement (zero KPS change). The difference in KPS improvement in the groups was significant ($p<0.01$). Activities of Daily Living Index indicated that poly I:poly $C_{12}U$ (Ampligen®) improved activity in all 13 life modules better than placebo. Exercise duration during treadmill testing was increased in the poly I:poly $C_{12}U$ treated group compared to placebo ($p < 0.04$). There was significantly less use of other medications in the poly I:poly $C_{12}U$ (Ampligen®) group, specifically less CNS specific drugs and also less pain relief medications. There was no difference in the total number of adverse events reported by subjects receiving poly I:poly $C_{12}U$, 706, as compared to placebo, 711 ($p > 0.90$). In addition, a shift table analysis of hematology, chemistry, and coagulation studies showed that there were no significant changes in laboratory test abnormalities among the poly I:poly $C_{12}U$ treated subjects when compared with the placebo group. Several subjects who received poly I:poly $C_{12}U$ had severe adverse events which were felt by the principal investigator to be related to other medical conditions. One individual experienced abdominal pain, tachycardia, and elevated hepatic enzymes 20 times normal. This subject resumed poly I:poly $C_{12}U$ treatment at a 50% dose reduction without any evidence of hepatic toxicity during an additional 12 months of therapy. Another subject with localized areas of swelling and itching was diagnosed with chilblains and also continued on poly I:poly $C_{12}U$ treatments. The results indicate that poly I:poly $C_{12}U$ is generally well tolerated and active in CFS/ME.

2.0 Objectives

2.1 To compare Exercise Tolerance Testing (ETT) in patients with CFS/ME treated with Ampligen® IV vs. placebo IV.

2.2 To compare Karnofsky Performance Score (KPS) in patients with CFS/ME treated with Ampligen® IV vs. placebo IV.

2.3 To compare Activities of Daily Living (ADL) in patients with CFS/ME treated with Ampligen® IV vs. placebo IV.

2.4 To compare Symptom Check List (SCL-90-R) scores in the Cognition and CFS/ME Dimensions in patients with CFS treated with Ampligen® IV vs. placebo IV.

2.5 To compare physical activity using an Activity Monitor (Actigraph) in patients with CFS/ME treated with Ampligen® IV vs. placebo IV.

2.6 To compare General Health Status (Short Form-36, Health Survey) in patients with CFS/ME treated with Ampligen® IV vs. placebo IV.

2.7 To compare the use of concomitant medications used to relieve symptoms of CFS/ME in patients treated with Ampligen IV vs. placebo IV.

2.8 To compare the frequency of hospital/emergency room admissions in CFS/ME patients treated with Ampligen IV vs. placebo IV.

2.9 To compare the incidence of side effects and toxicity in patients with CFS/ME treated with Ampligen® IV vs. placebo.

3.0 Study Design

This study will be a prospective, double-blind, randomized, placebo-controlled, equal parallel groups study conducted at up to fifteen (15) centers to compare the safety and efficacy of Ampligen® IV versus placebo IV in 230-240 patients with CFS/ME. Patients will be randomized and stratified to receive either Ampligen® intravenously or placebo (normal saline) intravenously. Fifty percent (50%) of the patients will be treated with Ampligen® IV and 50% of the patients will be treated with placebo IV.

Patients will be studied until 64 weeks (STAGES I plus II) have passed or until: 1) removed because of toxicity, 2) they withdraw voluntarily, 3) a change in the patient's medical condition makes continued participation unsafe, 4) the patient becomes non-compliant with the requirements of the protocol or 4) the Sponsor terminates the study.

The endpoints of the study are defined as follows:

3.1 Primary Endpoints:

3.1.1 Treadmill Exercise Tolerance Testing (ETT): Duration

3.1.2 Efficacy will be established by showing medically and statistically significant improvement in the primary endpoint, ETT, as defined in section 11.0 Statistical Considerations.

3.2 Secondary Endpoints:

3.2.1 Karnofsky Performance Score (KPS)
3.2.2 Activities of Daily Living (ADL)
3.2.3 SCL-90-R; Cognition and CFS Dimensions
3.2.4 Activity Monitoring
3.2.5 Short Form-36, Health Survey
3.2.6 Concomitant Medication used to relieve symptoms of CFS/ME
3.2.7 Frequency of hospital/emergency room admissions

Efficacy will be supported by statistically significant improvement of the secondary endpoints in the Ampligen® group compared to the placebo group.

4.0 Patient Selection

Two hundred thirty (230) to two hundred forty (240) CFS/ME patients with no prior exposure to Ampligen who meet the inclusion and exclusion criteria listed below will be randomized and admitted into Stage I of this protocol.

4.1 Inclusion criteria

4.1.1 A diagnosis of CFS, as defined by the Center for Disease Control (1988 CDC case definition) $\geq$ 12 months (Appendix D).

4.1.2 Age Range: $\geq$ 18 years old, $\leq$ 60 years old.

4.1.3 Males or non-pregnant, non-lactating females: Females must be of non-child bearing potential (either post-menopausal for two (2) years or surgically sterile including tubal ligation) or using an effective means of contraception (birth control pills, intrauterine device, diaphragm). Females who are less than two (2) years post-menopausal, those with tubal ligations and those using contraception must have a negative serum pregnancy test within the two (2) weeks prior to the first study medication infusion. Females of child bearing potential agree to use an effective means of contraception from four (4) weeks prior to the baseline pregnancy test until four (4) weeks after the last study medication infusion.

4.1.4 A reduced quality of life as determined by a documented KPS (Appendix E) of 40 to 60 on three (3) occasions, each at least 14 days apart, during the twelve (12) weeks immediately preceding the start of study drug infusions. The KPS must be rounded in increments of ten (10).

4.1.5 Ability to walk (minimum of 20 seconds) on the moving treadmill (grade = 0%; belt speed = 1 mph) on a minimum of two (2) occasions during the twelve (12) weeks immediately preceding study entry (See Appendix F).

4.1.6 Laboratory documentation (baseline or historical following onset of CFS/ME) of a negative ANA or a negative anti-ds (double-stranded) DNA, a negative Rheumatoid Factor, and an erythrocyte sedimentation rate (ESR).

4.1.7 Laboratory documentation that the patient is euthyroid (patients on thyroid replacement therapy must be on a stable dose during the eight (8) week washout period) based on a thyroid profile (T_4, T_3, TSH, T_3 uptake and Free T_4 index) performed during baseline.

4.1.8 Ability to provide written informed consent indicating awareness of the investigational nature of this study.

4.2 Exclusion Criteria

4.2.1 Inability to return to the investigator's site for scheduled infusions and evaluations (Sections 6.1, Dosing Schedule and 7.0, Evaluations and Assessments) during STAGES I and II of the study.

4.2.2 Chronic or intercurrent acute medical disorder or disease (Appendix D, Major Criteria, Number 2) making implementation or interpretation of the protocol or results difficult or unsafe.

4.2.3 Pregnant or lactating females (See 4.1.3).

4.2.4 Treatment with any of the following therapies within the eight (8) weeks immediately preceding the start of study baseline or during baseline: systemic glucocorticoids (ie, hydrocortisone, prednisone, etc.) or mineralocorticoids (ie, fludrocortisone (Florinef), etc.), interferons, interleukin-2, systemic antivirals (See Section 5.2), gamma globulin or investigational drugs and experimental agents not yet approved for use in the United States.

The patient must give written informed consent prior to discontinuation of any drugs listed under 4.2.4.

4.2.5 Prior participation in an Ampligen® study.

4.2.6 Medical necessity, as determined by the patient's private doctor or the principal investigator, to continue aspirin (ASA) or non-steroidal anti-inflammatory (NSAID) drugs for 20 consecutive days or for more than 10% of the study duration (i.e., 28 total days for STAGE I and 17 total days for STAGE II).

4.2.7 Ability to exercise over 18 minutes during any of the baseline ETT procedures.

4.2.8 Evidence or history of any of the following, which are exclusion criteria for the Exercise Tolerance Test (ETT) See Appendix F.

a. Previous documented evidence of myocardial infarction or recent significant change in the resting EKG suggesting infarction or other acute cardiac events.

b. Current symptoms of coronary insufficiency (i.e. - angina pectoris and/or ST segment depression on EKG).

c. Evidence of uncontrolled atrial or frequent or complex ventricular ectopy, or myocardial conduction defect which would increase the risk of syncope (for example, second degree or higher A-V block).

d. History of congestive heart failure, suspected or known dissecting aneurysm, recent systemic or pulmonary embolus, severe valvular heart disease, ventricular aneurysm, active or suspected myocarditis or pericarditis, thrombophlebitis or intracardiac thrombi, or acute infection.

e. Evidence of moderate or severe obstructive pulmonary disease.

f. Resting diastolic blood pressure > 115 mm Hg or resting systolic blood pressure > 200 mm Hg.

g. Uncontrolled metabolic disease (e.g., diabetes, thyrotoxicosis, or myxedema.

h. Concurrent use of any beta blockers and/or bronchodilators which cannot remain at a stable dosage level during the eight (8) week washout period and continuing during baseline and STAGES I and II.

4.2.9 History of alcohol or other substance abuse within two (2) years before the onset of the chronic fatigue and/or at any time afterward.

4.2.10 History of suicidal ideation or a suicide attempt within two (2) years of baseline.

4.2.11 Any past or current diagnosis of a major depressive disorder with psychotic or melancholic features; bipolar affective disorders; schizophrenia of any subtype; delusional disorders of any subtype; dementias of any subtype; anorexia nervosa; or bulimia nervosa.

5.0 Concomitant/Prohibited Medications

5.1 Concomitant Medications

Interactions between Ampligen® and other medications have not been completely evaluated. There are, therefore, potential risks associated with the concomitant use of medications with Ampligen® and their use should be closely monitored. All concomitant medications, their indications, and whether the use was related to a CFS/ME symptom will be recorded in the patient's medical record and case report form.

5.2 Prohibited Medications

The following drugs are prohibited from eight (8) weeks prior to the start of baseline until study termination: systemic glucocorticoids (ie, hydrocortisone, prednisone, etc.) or mineralocorticoids (ie, fludrocortisone (Florinef), etc.), interferons, interleukin-2, systemic antivirals including Zovirax (acyclovir), Valtrex (valacyclovir), Virazole (ribavirin), anti-retrovirals, Vistide (cidofovir), Cytovene (ganciclovir), Foscavir (foscarnet), Famvir (famciclovir), Flumadine (rimantadine), Symmetrel (Amantadine), and Kutapressin; gamma globulin, Ergamisol (levamisole), or investigational drugs and experimental agents not yet approved for use in the United States.

Use of aspirin (ASA) or non-steroidal anti-inflammatory drugs (NSAIDS) is limited during study treatment to less than 20 consecutive days or 10% of the study (28 total days for STAGE I).

The use of prohibited medication(s) is a violation of this protocol which could result in the Sponsor removing the patient from the study.

Patients on thyroid replacement therapy, beta blockers, and bronchodilators must be on a stable dose during the eight week washout period and continuing during baseline and STAGES I and II. Due to the decreasing availability of synthroid, patients on synthroid who are no longer able to obtain synthroid, may be switched to an equivalent thyroid hormone (levothyroxine) replacement therapy upon notification to and approval by the Medical Director of Hemispherx Biopharma, Inc.

A single short course of Flumadine or Amantadine (up to 100 mg twice a day for a maximum of 7 days) may be used for treatment of influenza A during STAGE I and again during STAGE II.

A single short course of gamma globulin or systemic steroids (up to a maximum of 14 days) may be used during STAGE I and again during STAGE II if medically indicated and approved in writing at the time by the Sponsor, Hemispherx Biopharma, Inc.

Investigators are discouraged from prescribing or recommending the use of any unapproved therapies for CFS/ME during the AMP 516 study from eight (8) weeks prior to the start of baseline, including baseline, STAGE I, and to the termination of STAGE II.

6.0 Study Medication

6.1 Dosing Schedule

Patients will be stratified and randomized to receive either Ampligen® or placebo (normal saline). Each patient will be assigned a unique allocation number which will appear on all study drug supplied for that patient during the 40 week double-blind phase. In order to maintain blinding of the study by minimizing the chance of a flushing and/or myalgic reaction to the study medication, infusion rates will be as follows for all patients:

INFUSION DOSE NUMBERS	INFUSION TIME
1, 5, 81 and 85	60 ± 5 minutes
2, 6, 82 and 86	45 ± 5 minutes*
3, 4, 7-80, 83, 84 and 87-128	35 ± 5 minutes*

* If prior infusion was well tolerated

During STAGE I, patients randomized to the Ampligen® arm will receive **Ampligen® 200 mg (80 ml) IV twice weekly** for the first four (4) doses (Weeks 1 and 2). Patients randomized to the placebo arm will receive an equivalent volume **(80 ml) of normal saline twice weekly** for the first four (4) doses (Weeks 1 and 2).

Starting with dose 5 (Week 3), patients randomized to the Ampligen® arm will receive **Ampligen® 400 mg (160 ml) IV twice weekly** through Week 40. Starting with dose 5 (Week 3), patients randomized to the placebo arm will continue to receive an equivalent volume **(160 ml) of normal saline IV twice weekly** through Week 40.

During STAGE II, all patients, regardless of treatment received (Ampligen® or placebo) during STAGE I, will receive **Ampligen® 200 mg (80 ml) IV twice weekly** for the first four (4) doses (#81, 82, 83 and 84)(Weeks 1 and 2). Starting with the first dose (#85) of Week 3, all patients will receive **Ampligen® 400 mg (160 ml) IV twice weekly** for the duration of their participation in this study.

During STAGES I and II, the first infusion at each dose level will be given over 60 ± 5 minutes; if well tolerated without significant infusion related side effects (e.g., flu-like symptoms), the next infusion will be over 45 ± 5 minutes; if well tolerated, all remaining infusions will be given over 35 ± 5 minutes. If significant infusion related side effects occur, the duration of the infusions will not be decreased until it is well tolerated. If significant infusion related side effects occur during the study, the infusion rate should be decreased before dosage modifications are considered.

6.2 Dosage Modification

Patients who experience any grade 3 or 4 drug-related toxicity will have the study medication discontinued unless the investigator can reliably exclude the role of the test drug (i.e. the relationship to study medication is "no"). The reason(s) for excluding the contribution of the study drug to the adverse event(s) must be documented by the investigator, and the medical monitor of the study must be notified. The study medication will be withheld until the toxicity abates to grade 1 or 0. Dosing will then be restarted at a 50% dose reduction. After 4 weeks of reduced dosing an attempt will be made to dose escalate these patients by increasing the dose to 75% of full dose for two weeks, and then 100% of full dose thereafter as long as patients do not experience a grade 3 or higher toxicity. However, if patients cannot tolerate the escalated dose, they will be maintained on the study at the 50% reduced dose.

Patients will remain on study for a total of 64 weeks (STAGE I plus STAGE II) or:

a. until an Ampligen® dose-limiting toxicity ("3" severe or "4" life-threatening) occurs (See Dosage Modification Section 6.2 above). Toxicity Grading may be found in Appendix A ("Toxicity Criteria"). Non-dose limiting toxicity ("1" mild or "2" moderate) will not necessitate the discontinuance of Ampligen® whereas dose-limiting toxicity ("3" severe or "4" life-threatening) which does not abate to grade 1 or less or recurs after a 50% dose reduction will be sufficient cause to terminate the patient from study;

b. the patient withdraws voluntarily from the study;

c. a change in the patient's medical condition makes continued participation unsafe;

d. the patient becomes non-compliant with the requirements of the protocol including > 10% total missed doses or > 2 consecutive missed doses;

e. the Sponsor terminates the study.

6.3 Drug Preparation

One to two individuals within each center will be selected to prepare the study medications for infusion.
The Ampligen® Administration Manual entitled "Procedures For Receiving, Storing, and Using Ampligen (Poly I:Poly $C_{12}U$) Liquid Solutions" contains specific directions for preparation of poly I:poly $C_{12}U$ for intravenous infusion. Forms which capture information regarding drug accountability, storage conditions and formulation will need to be completed on an ongoing basis for the duration of the study.

The use of plastic infusion bags for storing and preparing Ampligen® is specifically prohibited.

6.4 Drug Supplies

The Investigational Drug intravenous solutions will be provided in glass bottles containing poly I:poly $C_{12}U$ 200 mg (80 ml) or 400 mg (160 ml) per bottle, or placebo with a matching volume of normal saline. The poly I:poly $C_{12}U$ is dissolved in a sterile, preservative free, physiological salt solution (See Ampligen® Administration Manual).

The drug label will consist of two parts, one part will be separated at the perforations from the bottle and placed in the patient's case report form on the drug label page. The patient's identification code and date of administration will be written on this portion the label at the time of study medication infusion.

6.5 Blinding Procedures

The glass bottles utilized will be identical so that neither the patient, investigator, nor the nursing staff will be aware of the identity of the study medication administered. Each bottle is covered on its sides with an opaque colored tape to obscure the contents from visual inspection.

Patient treatment assignments will be maintained by the Sponsor. This information will only be provided to the investigator should a documented medical emergency situation arise in which knowledge of the treatment is required for medical management of the patient; in this instance, a written record of the conversation will be retained.

7.0 EVALUATIONS AND ASSESSMENTS

During each stage of the study, patients will have the evaluations and assessments as scheduled below.

7.1 Evaluation Methods (see Appendix B for Time Event Schedule)

a. Medical History and CFS/ME Diagnosis
A complete medical history will be conducted during baseline (within the twelve (12) weeks immediately preceding the start of study drug infusions) to determine eligibility for the study. The medical history will confirm a diagnosis of CFS, as defined by the CDC (Appendix D) for ≥ 12 months.

b. Physical Examination
A complete physical examination will be performed by the investigator during baseline, every eight (8) weeks during STAGES I and II, and at termination of STAGES I and II or early termination of STAGES I or II.

c. Assessment of CFS/ME Signs and Symptoms
An assessment of CFS signs and symptoms will be performed during baseline, every eight (8) weeks during STAGES I and II, and at termination of STAGES I and II or early termination of STAGES I or II. See Appendix G.

d. Karnofsky Performance Score (KPS)
KPS will be determined during baseline (3 times, each at least 14 days apart), every four (4) weeks during STAGES I and II, and at termination of each stage or early termination of STAGES I or II. During STAGES I and II of the study when the patient is returning twice weekly for study drug, the KPS will be determined at the time of the first visit for that week.[1] Note: The Week 20 KPS/ADL assessments may be done during weeks 19 or 20. See Appendix E.

e. Activities of Daily Living (ADL)
The ADL questionnaire will be completed by the patient (with assistance from his/her significant other, if necessary) in the presence of a health care professional during baseline (3 times, each at least 14 days apart), every four (4) weeks during STAGES I and II, and at termination or early termination of STAGES I or II. During STAGES I and II of the study when the patient is returning twice weekly for study drug, the ADL will be determined at the time of the second visit for that week.[2] Note: The Week 20 KPS/ADL assessments may be done during weeks 19 or 20.

f. Sera for Frozen Storage
A 20 ml blood sample will be obtained for serum collection and storage by Lab Corp on all patients during baseline (2 times, at least 14 days

---

[1] If a patient fails to return to the Investigator for a scheduled visit for KPS determination during any week of the study, KPS will be determined on the next visit.

[2] If a patients fails to return to the Investigator for a scheduled visit for ADL completion during any week of the study, ADL will be completed on the next visit.

apart), once every eight (8) weeks during STAGES I, II, and at termination of each stage or early termination of STAGES I or II. The sera will be shipped frozen on dry ice and stored frozen by Lab Corp at -70°C for possible future testing.

g. <u>Pregnancy Testing</u>
A standard HCG pregnancy test will be performed on every female patient of child-bearing potential within the two (2) weeks immediately preceding the start of study drug infusions, every eight (8) weeks during STAGES I and II, and at termination or early termination of STAGES I or II.

h. <u>Chemistry Panel</u>
The chemistry panel will consist of serum creatinine, electrolytes, calcium, phosphate, glucose, blood urea nitrogen, uric acid, cholesterol, total protein, albumin, bilirubin [total], alkaline phosphatase, lactic dehydrogenase, SGOT (AST), SGPT (ALT). The chemistry panel will be performed during baseline (2 times, at least 14 days apart), at weeks 4, 8, 16, 24, 32 and at termination of STAGE I and at weeks 4, 8 and 16 during STAGE II, and at termination of STAGE II or early termination of STAGES I or II.

i. <u>Urinalysis</u>
Urinalysis will be performed during baseline, at weeks 8, 16, 24, 32 and at termination of STAGE I, and every eight (8) weeks during STAGE II, and at the termination of STAGE II or early termination of STAGES I or II.

j. <u>Hematology Panel</u>
The hematology panel will include hematocrit, hemoglobin, total WBC with differential including neutrophils, lymphocytes, monocytes, eosinophils, basophils, and platelet count. The panel will be performed during baseline (2 times, at least 14 days apart), at weeks 4, 8, 16, 24, 32 and at termination of STAGE I, at weeks 4, 8 and 16 during STAGE II, and at termination of STAGE II or early termination of STAGES I or II.

k. <u>Coagulation Panel</u>
The coagulation panel (PT and PTT) will be performed once during baseline, at weeks 4, 8, 16, 24, 32 and at termination of STAGE I, at weeks 4, 8 and 16 during STAGE II, and at termination of STAGE II or early termination of STAGES I or II.

l. <u>Exercise Tolerance Test (ETT) and Electrocardiogram (EKG)</u>
The treadmill ETT (see Appendix F) including a resting 12 lead electrocardiogram (EKG) and EKG monitoring during the ETT, will be performed during baseline (minimum 2 times, with at least 14 "rest" days between tests), once during Week 20 ± 2 and twice at termination during Weeks 34 to 39 (with at least 14 "rest" days between tests), or if patient termination is earlier (2 times, with at least

14 days between tests). A "rest" day is any day the ETT is not performed. When the two (2) baseline exercise tests differ by more than ± 10% of the maximum duration from their mean value, a third treadmill ETT and EKG will be performed at least 14 days from the second ETT. When three (3) baseline treadmill ETTs are performed, only the two (2) closest values will be used for data analysis. The ETT and EKG will be performed on day 8 of each 15 day activity monitoring cycle. A single ETT and EKG will be performed during weeks 21 to 23 of STAGE II on day 8 of the 15 day activity monitoring cycle.

m. Activity Monitoring with Patient Activity Diary

A physical activity monitor (Actigraph) will be assigned to patients with data collection being completed over 15 day intervals (cycles). The Activity Monitor will be worn on a belt at the waist. Data from two separate 15 day recording cycles will be collected at baseline, then once at week 20 ± 2 and twice at termination of STAGE I. During baseline, during weeks 20 ± 2 and at termination the collection of activity data will include the entire seven (7) (i.e. 24 hours each day) days before each Treadmill Exercise Tolerance Test (ETT), the day the ETT is performed, and the entire seven (7) days after the ETT. Each cycle will be 15 days long with the ETT performed on day 8. The same Activity Monitor (identified by serial #) will be utilized (unless impossible for technical reasons, including scheduling difficulties) for all testing performed on any particular patient throughout the study. If activity monitoring data is not obtained because of a technical problem with the monitor, or other reason(s), the Activity Monitor testing will be repeated, as soon as possible, but in this case an ETT will not be required on day 8. If a third ETT is required at baseline, activity monitoring will not be performed if two adequate 15 day collections have already been completed. A single 15 day data collection will be performed during weeks 20 to 23 of STAGE II. In order to record each entire 15 day period (7 days before and after each ETT), the Activity Monitor will usually need to be placed on the subjects 1 to 3 days before, (and removed 1 to 3 days after) the 15 day cycle begins (or ends).

Patients will be required to record every sustained activity of >5 minutes duration while the activity monitor is worn.

n. Chest X-Ray (CXR)

A CXR (PA and Lateral) will be performed during baseline, Week 40 or at early termination (if prior to Week 40).

o. Health Resource Utilization/Employment Survey

The Health Resource Utilization/Employment Survey will be completed at baseline and every four (4) weeks during STAGES I and II and at early termination of STAGES I or II.

p. Symptoms Check List-90-Revised (SCL-90-R)
The SCL-90-R will be completed during baseline, at weeks 8, 16, 24, 32 and at termination of STAGE I, and every eight (8) weeks during STAGE II and at termination of STAGE II or early termination of STAGE I or II.

q. Short Form-36, Health Survey (SF-36)
The SF-36 will be completed during baseline, at weeks 8, 16, 24, 32 and at termination of STAGE I, and every eight (8) weeks during STAGE II and at termination of STAGE II or early termination of STAGE I or II.

r. Thyroid Profile
A thyroid profile (T_4, T_3, TSH, T_3 uptake and Free T_4 index) will be done once at baseline, at weeks 8, 24, and at termination or early termination of STAGE I, and at week 8 and termination or early termination of STAGE II.

s. Drug Administration/Vital Signs
Study medication will be infused twice weekly in accordance with Section 6.0, Study Medication. With their first infusion, each patient should be placed on a Monday/Thursday or Tuesday/Friday infusion schedule. All deviations in the dosing schedule including missed doses are to be documented in the patient's medical record including the reason for the deviation. Vital signs (pulse, blood pressure, respiratory rate, and temperature) must be taken prior to and at the end of the study drug infusions.

t. Adverse Events Reporting
The presence or absence of Adverse Events will be assessed during every scheduled visit during the study and as needed between visits.

u. Concomitant Medications
Changes in concomitant medications will be assessed during every scheduled visit during STAGES I and II of the study and as needed between visits.

v. Study Blind Questionnaire
At the end of STAGE I patients and investigators will complete questionnaires to guess as to which therapy was given to each patient.

w. Immune Panel (optional)
RNAse L activity and/or molecular weight profile/ratio will be done at baseline, and at termination of STAGES I and II.

x. Locator Questionnaire
A questionnaire (See Appendix H) designed to facilitate long-term follow-up of study participants will be completed during Baseline and at the end of STAGE II, or at the time of an earlier termination.

7.2 Scheduling (See Appendix B).

a. Baseline (Must be performed during the twelve (12) weeks prior to starting therapy except Pregnancy test as noted below):

   i. Medical History and confirmation of a CFS Diagnosis
   ii. Physical Examination
   iii. Assessment of CFS/ME Signs and Symptoms
   iv. KPS (3 times, each at least 14 days apart). Note: KPS and ADL should be completed on different clinical visits, see Sections 7.1d and e
   v. ADL (3 times, each at least 14 days apart)
   vi. Sera for Frozen Storage (2 times, at least 14 days apart)
   vii. Pregnancy Test (within the 2 weeks immediately preceding the start of study drug administration)
   viii. Chemistry Panel (2 times, at least 14 days apart)
   ix. Urinalysis
   x. Hematology Panel (2 times, at least 14 days apart)
   xi. Coagulation Panel
   xii. ETT and EKG (minimum 2 times, with at least 14 "rest" days between tests)
   xiii. Activity Monitoring: two 15 day collection periods centered around each ETT.
   xiv. CXR: PA and Lateral
   xv. Health Resource Utilization/Employment Survey
   xvi. SCL-90-R
   xvii. Thyroid Profile
   xviii. SF-36
   xix. Immune Panel (optional)

b. Two (2) times weekly during STAGES I and II
   i. Drug Administration Record/Vital Signs
   ii. Adverse Events
   iii. Concomitant Medications

c. Week 4 of STAGES I and II
   i. Chemistry panel
   ii. Hematology panel
   iii. Coagulation panel

d. Every 4 weeks during STAGES I and II
   i. KPS
   ii. ADL
   iii. Health Resource Utilization/Employment Survey

e. Week 20 ± 2 (STAGE I)

- i. ETT and EKG (To be done on day 8 of 15 day Activity Monitoring Cycle)
- ii. Activity Monitoring (15 day cycle with ETT on day 8)

f. Every 8 Weeks (during STAGES I and II)

- i. Physical Examination
- ii. Assessment of CFS/ME Signs and Symptoms
- iii. Sera for Frozen Storage
- iv. Pregnancy Test
- v. Chemistry Panel
- vi. Urinalysis
- vii. Hematology Panel
- viii. Coagulation Panel
- ix. SCL-90-R
- x. SF-36

g. Week 8 (STAGES I and II) and Week 24 (STAGE I)

- i. Thyroid Profile

h. Termination: STAGE I or early termination

- i. Physical Examination
- ii. Assessment of CFS/ME Signs and Symptoms
- iii. KPS
- iv. ADL
- v. Sera for Frozen Storage
- vi. Pregnancy Test
- vii. Chemistry Panel
- viii. Urinalysis
- ix. Hematology Panel
- x. Coagulation Panel
- xi. ETT and EKG (2 times during weeks 34 to 39 with at least 14 "rest" days between each ETT). In the event of an early termination which occurs at least four weeks after a previous ETT evaluation, the two sequential activity monitoring/ETT cycles would be started as soon as possible. No additional ETT evaluations would be performed if an early termination occurs less than four weeks from a previous ETT evaluation, or if patient has received less than four weeks of drug treatment. The ETT and EKG must be performed on day 8 of each activity monitor recording cycle.
- xii. CXR: PA and Lateral (not required if patient has received less than eight (8) weeks of drug treatment)
- xiii. SCL-90-R
- xiv. SF-36
- xv. Thyroid Profile
- xvi. Drug Administration Record/Vital Signs

xvii. Adverse Events.
xviii. Concomitant Medications
xix. Activity Monitoring (Two 15 day activity recording cycles with each cycle centered around the termination ETTs, i.e. on day 8 of the 15 day cycle)
xx. Study Blind Questionnaire (if patient completes STAGE I)
xxi. Health Resource Utilization/Employment Survey
xxii. Immune Panel (optional)

i. Termination: STAGE II or early termination
i. Physical Examination
ii. Assessment of CFS/ME Signs and Symptoms
iii. KPS
iv. ADL
v. Sera for Frozen Storage
vi. Pregnancy Test
vii. Chemistry Panel
viii. Urinalysis
ix. Hematology Panel
x. Coagulation Panel
xi. SCL-90-R
xii. SF-36
xiii. Drug Administration Record/Vital Signs
xiv. Adverse Events
xv. Concomitant Medications
xvi. Activity Monitoring (one 15 day cycle centered around ETT)
xvii. Thyroid Profile
xviii. ETT and EKG (for early termination the ETT and EKG are not required if patient has received less than four (4) weeks of drug treatment)
xix. Health Resource Utilization/Employment Survey
xx. Immune Panel (optional)

j. Follow-up
The following six questionnaires will be utilized to secure follow-up information on all patients who withdraw early (early termination) from the study (ie. prior to completion of STAGE II) and all patients who complete the study including patients who continue to receive Ampligen while enrolled in AMP 511:
i. SCL-90-R
ii. SF-36
iii. ADL
iv. KPS Questionnaire
v. Assessment of CFS/ME Signs and Symptoms
vi. Health Resource Utilization/Employment Survey

At Baseline and early termination or at the completion of STAGE II, patients will be requested to complete a Locator Questionnaire (see Appendix H) which will provide information concerning the addresses and telephone numbers of the patient and friends and family members, who would likely know the

whereabouts of the patient in the event that the patient relocates from their current address. This information will be kept by the investigator in a special Locator Questionnaire File to be utilized only if necessary to help locate the patient at some later point in time.

The six questionnaires will be mailed by the investigator to the patients along with instructions for completion and with a pre-stamped and addressed envelope to be used to return the completed questionnaires to Hemispherx Biopharma.

The patients will be identified on the questionnaires by code only.

Patients who terminated early will be requested to complete the same six questionnaires at the date (± 4 weeks) corresponding to Week 20 and Week 40 of STAGE I and Week 24 of STAGE II.

Patients who complete STAGE I will be requested to complete the six questionnaires at 6, 12, 18 and 24 months (± 4 weeks) following completion of STAGE II, or following an earlier termination from the study. If a patient enters AMP-511 and completes one of the required follow-up questionnaires as part of the AMP-511 study, that data will be sufficient as long as the date of completion of the questionnaires is within ±4 weeks of the time limits set above.

8.0 Management of Intercurrent Events

8.1 Departure From Protocol

If a circumstance arises which may justify a departure from protocol for an individual patient, the investigator should contact the Sponsor for a joint decision as to whether the protocol may be modified for that patient, or whether the subject should be prematurely discontinued from the study. The reason for such a joint decision will be documented in writing and may require Institutional Review Board approval.

8.2 Adverse Events

An adverse event is defined as any untoward occurrence whether or not considered to be study drug related. Adverse events observed by the investigator and/or reported by the patient include new symptoms or medical conditions, exacerbations of preexisting illnesses or symptoms, common ailments such as headaches, upper respiratory infections and signs or symptoms.

Adverse events will be collected from the start of STAGE I until study termination. The investigator will monitor each patient for evidence of drug intolerance/toxicity and for the development of clinical and/or laboratory evidence of an adverse event. The investigator is to assess whether the adverse event has any relationship to the study medication. All adverse events should be followed until resolution. The minimum information required for each adverse event includes: date of onset, duration, severity, frequency, etiology,

course and treatment, relationship to study medication, and outcome of the event. Laboratory values are to be reported if the reaction is considered possibly, probably, or definitely drug related or when the laboratory values are relevant to the adverse event. Guidelines for determining severity and relationship to study medication are as follows:

NO	No drug relationship exists
REMOTE	Less than two (2) of the four (4) criteria are met
POSSIBLE	At least two (2) of the criteria are met
PROBABLE	At least three (3) of the criteria are met
DEFINITE	Drug caused the event.

CRITERIA:

1. A temporal relationship exists between the event and the use of the drug.
2. Re-administration of study medication(s) results in reappearance or worsening of the reaction.
3. Previous experience with the suspected or related compounds resulted in a similar reaction.
4. Event is not related to any concomitant disease, preexisting condition, other drug therapy, or environmental factors.

SEVERITY:

MILD - transient and easily tolerated
MODERATE - causes discomfort and interrupts normal activities
SEVERE - considerable interference with normal activities and may be incapacitating

8.3 Serious Adverse Events

A serious adverse event or reaction is any untoward medical occurrence that at any dose results in death, is life-threatening, requires inpatient hospitalization or prolongation of existing hospitalization, results in persistent or significant disability/incapacity, or is a congenital anomaly/birth defect. Important medical events that may not result in death, be life-threatening, or require hospitalization may be considered a serious adverse drug experience when, based upon appropriate medical judgment, they may jeopardize the patient or subject any may require medical or surgical intervention to prevent one of the outcomes listed in this definition. All serious adverse events shall be reported immediately to the Medical Director (or his designee), of Hemispherx Biopharma, and will be followed by a written narrative report by the investigator outlining the details of the adverse event. This report will be sent to Hemispherx Biopharma within three (3) working days. Serious adverse events shall also be documented in the case report form.

If no adverse events occur, this should be noted on the case report form.

All Serious Adverse events require written IRB notification.

9.0 Reasons for Discontinued Participation

Patients who fail to comply with the requirements of the study (Sections 5.2, Prohibited Medications; 6.1, Dosing Schedule and 7.0, Evaluations and Assessments) will be withdrawn from analysis and declared dropouts. Reasons for dropouts may include the following:

* Voluntary patient withdrawal.
* Failure to comply with the requirements of the study (i.e., failure to return for visits) or use of prohibited concomitant medications.
* Significant intercurrent illness or surgery, as determined by the investigator which prevents the patient from taking the study medication, or which requires administration of drugs disallowed in this study.
* Toxicity grading of 3 or 4 (see Appendix A and Section 6.2 Dosage Modification)

10.0 Administrative Aspects

Except in a medical emergency, deviation from this protocol should not be made other than as part of a protocol amendment agreed upon with the Sponsor.

10.1 Institutional Review Board (IRB) and Informed Consent

The investigator's IRB will review and approve the study protocol and patient Informed Consent document. After approval by the committee, the following will be sent to the Sponsor before the study commences:

a. the IRB Approval Letter.
b. the approved patient Informed Consent document
c. a list of IRB members, their representative capacity, and their affiliation with the institution (or documentation sufficient to ensure compliance - General Assurance number).

Each patient must give written informed consent to the investigator prior to the performance of any study related procedures including prohibited medication washout. The original signed consent form will be filed in the Investigator Document File binder. A copy of the signed consent form will be maintained on file in the patient's permanent medical records. Another copy will be given to the patient. The original signed consent form may be inspected at the Sponsor's/FDA's request.

10.2 Study Commencement and Discontinuation

Upon satisfactory receipt of all necessary paperwork, the Sponsor will arrange that all study material be delivered to the study site and that a mutually convenient appointment is set up for the conduct of an initiation visit. Patient entry must not begin until this initiation visit has been made. At this meeting, all personnel expected to be involved in the conduct of the study will undergo an orientation to include review of study protocol, instruction for case report form completion and overall responsibilities including those for drug accountability and study file maintenance.

10.3 Data Recording

Case report forms will be provided by the Sponsor for the collection of all study data. A copy is to be retained in the investigator's files. The forms should be printed or written legibly, using a typewriter or black ballpoint pen. They should be completed in a timely manner and every effort to have forms completed and up-to-date should be made in anticipation of a visit by the Sponsor.

Case report form completion may be delegated to other study personnel; however, the sponsor must be apprised in writing of the name of such persons and the scope of their authority. It is the obligation of the investigator to review each page of the case report form. If, for any reason, certain data are lacking to complete an individual case report form, a written statement will be provided by the investigator explaining the reasons for the lack of data.

10.4 Monitoring of Study

The investigator will permit a representative of Hemispherx Biopharma or a contract research organization (CRO), or the FDA to inspect all case report forms and the study patient's medical records (including office and hospital) at regular intervals throughout the study. These inspections are for the purpose of verifying adherence to the protocol and the completeness and exactness of the data being entered on the case report forms.

10.5 Drug Accountability

The investigational materials are to be prescribed by only the principal investigator or the sub-investigators named in FDA Form 1572. Under no circumstances will the investigator allow the investigational drug to be used other than as directed by the protocol.

Accurate records must be maintained accounting for the receipt of the investigational materials and for the disposition of the material. This should consist of a dispensing record including the identification of the person to whom the drug is dispensed, the quantity and the date of dispensing, and any unused drug returned. This record is in addition to any drug accountability information recorded on the case report forms. At the termination of the study or at the request of the Sponsor, the investigator must return any unused supplies to the Sponsor.

10.6 Investigator's Final Report

Upon completion or termination of the study, the principal investigator will submit a final written report as required by Federal Regulations. The report should be submitted to the sponsor and to the IRB within 90 days of completion or termination of the study.

The report should include, but not be limited to, a description of the study objectives, methodology (including any deviations from protocol), results and conclusions regarding attainment of study objectives. All significant adverse

experiences must be described, with the principal investigator's opinions as to whether they were or were not related to study drug administration.

10.7 Disclosure of Data

All information obtained during the conduct of this study will be regarded as confidential. Disclosures of results of the investigation for publication or by oral or poster presentation shall not be made earlier than 30 days after submission of the proposed materials to the Sponsor for inspection, unless the Sponsor consents to earlier disclosure. The principal investigator will take appropriate cognizance of Sponsor's suggestions before disclosure for publication or presentation consistent with protection of the Sponsor's right in its confidential data.

11:0 Statistical Considerations

11.1 Endpoints

The main aim of this protocol is to determine whether the study medication in the dosage given improves performance and alleviates symptomatology associated with CFS/ME. The primary clinical endpoint of the study is:

Treadmill Exercise Tolerance Testing (ETT): Duration

Efficacy will be established by showing medically and statistically significant improvement, as defined below, of the primary endpoint, ETT.

11.1.1 Primary Endpoint:

1. Treadmill Exercise Tolerance Testing (ETT): Duration

2. Efficacy will be established by showing a medically significant increase ($\geq$ 6.5%) in mean exercise duration (baseline compared to week 40) that is statistically significant ($p \leq 0.05$) using analysis of covariance of $\log_{10}$ transformed data with Baseline ETT duration as covariate. Note: Analysis of past ETT data suggests that the $\log_{10}$ transformation will result in compliance of the usual assumptions required for the t-test. For each patient the mean Baseline exercise duration will be calculated as described in Section 7.1.I, "Exercise Tolerance Test (ETT) and Electrocardiogram (EKG)" (Note: the Baseline ETT duration is actually the mean of the two closest Baseline measurements). For each patient the $\log_{10}$ of the mean Baseline exercise duration will be subtracted from the $\log_{10}$ of the Termination ETT duration (mean of two ETT determinations obtained during weeks 34 to 39). An analysis of covariance will be performed using Baseline exercise duration as a covariate. Analyzing the difference between log durations is equivalent to analyzing percent change from Baseline, since the difference between logs is equivalent to the log of the ratio and the ratio is functionally equivalent to percent change. We will also analyze the median absolute changes in ETT duration between Termination and Baseline using the Mann Whitney test.

11.1.2 Secondary Endpoints:

1. Karnofsky Performance Score (KPS)

Since the KPS scale is a discontinuous variable and the distribution of KPS does not follow a normal distribution, the median KPS score represents the most appropriate statistical parameter to compare KPS scores in clinical trials. A 10 point increase in median KPS will be considered a medically significant change, and efficacy will be supported by a statistically significant increase in the proportion of patients receiving Ampligen® who increase median KPS by ≥ 10 points (Week 40 vs. Baseline) compared to patients receiving placebo (Fisher's Exact Test). We will also analyze the mean and median absolute changes in KPS between each KPS recorded during the study (every 4 weeks) and baseline using the student-t (ANOVA) and Mann Whitney tests.

2. Activities of Daily Living (ADL)
3. Symptoms Check List-90-Revised (SCL-90-R); CFS Dimension and Cognition
4. Activity Monitoring
5. Short Form-36, Health Survey; Vitality and General Health Perceptions
6. Concomitant Medications used to relieve symptoms of CFS/ME
7. Frequency of hospital/emergency room admissions

Treatment Effects for other secondary endpoints
A Mann-Whitney or Wilcoxon rank-sum test with the student t-test will be used to assess the probability that changes observed were due to treatment effects rather than caused by chance variation in each secondary endpoint. A paired t-test will reflect the individual change and the sample change and the probability that this change is caused by random factors. These tests will be performed comparing baseline to successive study intervals, out to the study conclusion at forty weeks. Statistical significance will be established with p-values $\leq .05$ at the study conclusion of forty weeks. To further distinguish and separate selection impact from treatment effect, ANCOVA's will be done with baseline secondary endpoints as covariate.

11.2 Sample Size

With 100 persons per treatment group, a power of ≥ 80% and Type I error of 5% (two-sided) will be achieved. This will enable us to detect a significant increase (Fisher's exact test) in the proportion of CFS patients receiving poly I:poly $C_{12}U$ that improve Karnofsky Performance Score (KPS) by ≥ 20% compared to placebo.

11.3 Randomization

Subjects will be stratified according to treadmill duration (≤ 9 minutes vs. > 9 minutes). The subjects will be randomized to receive poly I:poly $C_{12}U$ or placebo according to randomization schedules utilizing a block design. In this approach, separate randomization schedules will be used for the two treadmill duration strata at each site.

The Mann-Whitney or Wilcoxon rank-sum test will be used with a student t-test to demonstrate that randomization to placebo and treatment groups has eliminated bias in the selection process and that the null hypothesis (H_0) at baseline is true. All endpoints will be examined in the placebo and treatment groups with these tests.

A Chi-Square test for subset stratification (treadmill duration) by treatment/placebo will reveal any inadvertent disproportion of stratification in either arm.

11.4 Subset Analyses

Subset analyses will be performed with regard to the stratification parameter treadmill duration.

11.5 Dropouts and Dose-Reductions

An additional 30 to 40 patients will be entered onto the study to compensate for an expected maximum dropout rate of 15-20%. Therefore, the total number of patients will be 230 to 240. Analysis of efficacy will take dropouts into account in two different ways. First, analysis of only evaluable patients who completed STAGE I (40 weeks) will be done. Secondly, analysis (of intent to treat) will be done including all patients who received study drug and performed the study parameter during STAGE I. Data from the last study parameter performed during STAGE I will be carried forward. With regard to patients who complete STAGE I at a reduced dose, data analysis will be performed two ways (both including and excluding patients who completed the study at the dose reduction).

		0	1	2	3	4
Leukopenia	WBC x 10^{-3}<br>NEUT x 10^{-3}	≥4.5<br>≥1.9	3.0-<4.5<br>1.5-<1.9	2.0-<3.0<br>1.0-<1.5	1.0-<2.0<br>0.5-<1.0	<1.0<br><0.5
Thrombocytopenia	Plt x 10^{-3}	>130	90-<130	50-<90	25-<50	<25
[illegible]ia	Hgb gm %<br>Hct %<br>Clinical	≥11<br>≥32	9.5-10.9<br>28-31.9	<9.5<br><28<br>Symptoms of anemia	Require transfusions	
Hemorrhage		None	Minimal	Moderate-not debilitating	Debilitating	Life Threatening
Infection		None	No active treatment	Requires active treatment	Debilitating	Life Threatening
GU*	BUN mg %<br>Creatinine<br>Protein<br>Hematuria	≤20<br>≤1.2<br>Negative<br>Negative	21-40<br>1.3-2.0<br>1+<br>Micro-Cult-positive	41-60<br>2.1-4.0<br>2+-3+<br>Gross-Cult positive	>60<br>>4.0<br>4+<br>Gross plus Clots	Symptomatic Uremia<br>With obst uropathy
Hepatic**	SGOT<br>Alk Phos<br>Bilirubin<br>Clinical	<1.5 x nl<br><1.5 x nl<br><1.5 x nl	1.5-2 x normal<br>1.5-2 x normal<br>1.5-2 x normal	2.1-5 x normal<br>2.1-5 x normal<br>2.1-5 x normal	>5 x normal<br>>5 x normal<br>>5 x normal<br>Pre-coma	Hepatic coma
N & V		None	Nausea	Nausea and Vomiting controllable	Vomiting intractable	
Diarrhea		None	No dehydration	Dehydration	Grossly bloody	
Pulmonary***	Clinical	normal	Mild or transient symptoms with <25% decrease in DCO or VC (if measured)	Moderate Symptoms with 25-50% decrease in DCO or VC (if measured)	Severe Sx-intermittent O_2 or >50% decrease in DCO or VC	Assisted vent or continuous O_2
Cardiac		normal<br>normal	ST-T changes<br>Sinus tachycardia > 110 at rest	Atrial arrhythmias<br>Unifocal PVC's	Mild CHF<br>Multifocal PVC's<br>Pericarditis	Severe or refractory Congestive Heart Failure<br>Ventricular tachycardia<br>Tamponade
Neuro	PN	None	Decreased DTR's<br>Mild paresthesias<br>Mild Constipation	Absent DTR's<br>Severe paresthesias<br>Severe Constipation<br>Mild weakness	Disabling sensory loss<br>Severe PN pain Obstipation<br>Severe weakness<br>Bladder dysfunction	Respiratory dysfunction secondary to weakness<br>Obstipation requiring surgery<br>Paralysis - confining pt to bed/wheelchair
	CNS	None	Mild anxiety<br>Mild depression<br>Mild headache<br>Lethargy	Severe anxiety<br>Moderate depression<br>Moderate headache<br>Somnolence<br>Tremor<br>Mild hyperactivity	Confused or manic<br>Severe depression<br>Severe headache<br>Cord dysfunction<br>Confined to bed due to CNS dysfunction	Seizures<br>Suicidal<br>Coma
Skin & Mucosa		normal	Transient erythema<br>Pigmentation atrophy	Vesiculation<br>Subepidermal fibrosis	Ulceration<br>Necrosis	
Oral	Stomatitis	normal	Soreness	Ulcers - can eat	Ulcers - cannot eat	
Alopecia		None	Alopecia-mild	Alopecia-severe		
Allergy		None	Transient rash<br>Drug Fever (≤38°C, ≤100.4°F)	Urticaria<br>Drug fever >38°C (>100.4°F)<br>Mild bronchospasm	Serum sickness<br>Bronchospasm - requiring parenteral medications	Anaphylaxis
Fever****		≤38°C	≤39°C	>39°C	>40.3°C	Fever induced hypotension
Local Toxicities		None	Pain	Pain- Phlebitis	Ulceration	
Coagulation	PT<br>PTT	within normal limits<br>within normal limits	1.01-1.25 x normal<br>1.01-1.66 x normal	1.26-1.50 x normal<br>1.67-2.33 x normal	1.51-2.00 x normal<br>2.34-3.00 x normal	> 2.00 x normal<br>>3.00 x normal

1. The toxicity grade should reveal the most severe degree occurring during the evaluation period not an average.
2. When two criteria are available (or similar toxicities e.g., leukopenia, neutropenia the more severe toxicity grade should be used).
3. Toxicity grade = 5 if that toxicity caused the death of patient.

* Urinary tract infection should be graded under infection not GU. Hematuria resulting from thrombocytopenia is graded under hemorrhage.
** Viral hepatitis should be recorded as infection rather than liver toxicity.
*** Pneumonia is considered infection and not graded as pulmonary toxicity unless felt to be resultant from changes directly induced by treatment.
**** Fever felt to be caused by drug allergy should be graded as allergy. Fever due to infection is graded under infection only.

A[illegible]ENDIX B
TIME AND EV[illegible]SCHEDULE (STAGE I)
AMPLIGEN® CLIN[illegible]L PROTOCOL #AMP-516
PATIENTS WITH CFS/ME

		STAGE I: Double-blind, Randomized, Placebo Controlled (Weeks)															
Test Week No.	Baseline	1	2	3	4	5	6	7	8	9	10	11	12	13	14	15	16
Informed Consent[1]	X																
Medical Hx - CDC Dx/Locator Quest.[3]	X																
Physical Exam	X								X								X
CFS/ME Assessment S/S	X								X								X
KPS and ADL[2]	XXX				X				X				X				X
Sera Collection	XX								X								X
Pregnancy Test (Female)	X								X								X
Chemistry Panel	XX				X				X								X
Urinalysis	X								X								X
Hematology Panel	XX				X				X								X
Coagulation Panel	X				X				X								X
ETT and EKG	XX(X)																
Activity Monitor	XX																
Chest X-Ray	X																
Health Res. Util/Employ Survey	X				X				X				X				X
SCL-90-R & SF-36	X								X								X
Thyroid Profile	X								X								
Immune Panel (optional)	X																
Study Blind Questionnaire																	
Concomitant Meds	X	XX	XX	XX	XX	XX	XX	XX	XX	XX	XX	XX	XX	XX	XX	XX	XX
Drug Administration/Vital Signs		XX	XX	XX	XX	XX	XX	XX	XX	XX	XX	XX	XX	XX	XX	XX	XX
Adverse Events/Hosp.		XX	XX	XX	XX	XX	XX	XX	XX	XX	XX	XX	XX	XX	XX	XX	XX

1. Informed Consent must be signed prior to starting any prohibited drug washout.
2. The KPS will be determined at the time of the first visit for that week and the ADL will be completed at the time of the second visit for that week.
3. Locator Questionnaire is to be completed at Baseline and at time of an early termination.

A[illegible]ENDIX B
TIME AND EV[illegible]SCHEDULE (STAGE I)
AMPLIGEN® CLINI[illegible]AL PROTOCOL #AMP-516
PATIENTS WITH CFS/ME

	STAGE I: Double-blind, Randomized, Placebo Controlled (Weeks) con't															
Test Week No.	17	18	19	20	21	22	23	24	25	26	27	28	29	30	31	32
Informed Consent[1]																
Medical Hx - CDC Dx/Locator Quest.[3]																
Physical Exam								X								X
CFS/ME Assessment S/S								X								X
KPS and ADL[2]				X				X				X				X
Sera Collection								X								X
Pregnancy Test (Female)								X								X
Chemistry Panel								X								X
Urinalysis								X								X
Hematology Panel								X								X
Coagulation Panel								X								X
ETT and EKG				X												
Activity Monitor				X												
Chest X-Ray																
Health Res. Util/Employ Survey				X				X				X				X
SCL-90-R & SF-36								X								X
Thyroid Profile								X								
Immune Panel (optional)																
Study Blind Questionnaire																
Concomitant Meds	XX	XX	XX	XX	XX	XX	XX	XX	XX	XX	XX	XX	XX	XX	XX	XX
Drug Administration/Vital Signs	XX	XX	XX	XX	XX	XX	XX	XX	XX	XX	XX	XX	XX	XX	XX	XX
Adverse Events/Hosp.	XX	XX	XX	XX	XX	XX	XX	XX	XX	XX	XX	XX	XX	XX	XX	XX

1. Informed Consent must be signed prior to starting any prohibited drug washout.
2. The KPS will be determined at the time of the first visit for that week and the ADL will be completed at the time of the second visit for that week.
3. Locator Questionnaire is to be completed at Baseline and at time of an early termination.

A[illegible]ENDIX B
TIME AND EV[illegible]SCHEDULE (STAGE I)
AMPLIGEN® CLINI[illegible]AL PROTOCOL #AMP-516
PATIENTS WITH CFS/ME

	STAGE I: Double-blind, Randomized, Placebo Controlled (Weeks) con't							
Test Week No.	33	34	35	36	37	38	39	40[3]
Informed Consent[1]								
Medical Hx - CDC Dx/Locator Quest.[4]								
Physical Exam								X
CFS/ME Assessment S/S								X
KPS and ADL[2]				X				X
Sera Collection								X
Pregnancy Test (Female)								X
Chemistry Panel								X
Urinalysis								X
Hematology Panel								X
Coagulation Panel								X
ETT and EKG			X			X		
Activity Monitor			X			X		
Chest X-Ray								X
Health Res. Util/Employ. Survey				X				X
SCL-90-R & SF-36								X
Thyroid Profile								X
Immune Panel (optional)								X
Study Blind Questionnaire								X
Concomitant Meds	XX	XX	XX	XX	XX	XX	XX	XX
Drug Administration/Vital Signs	XX	XX	XX	XX	XX	XX	XX	XX
Adverse Events/Hosp.	XX	XX	XX	XX	XX	XX	XX	XX

1. Informed Consent must be signed prior to starting any prohibited drug washout.
2. The KPS will be determined at the time of the first visit for that week and the ADL will be completed at the time of the second visit for that week.
3. Week 40 assessments which cannot be completed during week 40 because of scheduling problems may be completed during week 41 with approval of sponsor.
4. Locator Questionnaire is to be completed at Baseline and at time of an early termination.

APPEN[illegible] B (continued)
TIME AND EVE[illegible]SCHEDULE (STAGE II)
AMPLIGEN® CLINICAL PROTOCOL #AMP-516
PATIENTS WITH CFS/ME

	STAGE II: Open Label Safety Evaluation (Weeks)																							
Test Week No.	1	2	3	4	5	6	7	8	9	10	11	12	13	14	15	16	17	18	19	20	21	22	23	24[4]
Informed Consent[1]																								
Medical Hx - CDC Dx/Locator Quest.[3]																								X[3]
Physical Exam								X								X								X
CFS/ME Assessment S/S								X								X								X
KPS and ADL[2]				X				X				X				X				X				X
Sera Collection								X								X								X
Pregnancy Test (Female)								X								X								X
Chemistry Panel				X				X								X								X
Urinalysis								X								X								X
Hematology Panel				X				X								X								X
Coagulation Panel				X				X								X								X
ETT and EKG																					X			
Activity Monitor																					X			
Chest X-Ray																								
Health Res. Util./Employ. Survey				X				X				X				X				X				X
SCL-90-R & SF-36								X								X								X
Thyroid Profile								X																X
Immune Panel (optional)																								X
Concomitant Meds	XX	XX	XX	XX	XX	XX	XX	XX	XX	XX	XX	XX	XX	XX	XX	XX	XX	XX	XX	XX	XX	XX	XX	XX
Drug Administration/Vital Signs	XX	XX	XX	XX	XX	XX	XX	XX	XX	XX	XX	XX	XX	XX	XX	XX	XX	XX	XX	XX	XX	XX	XX	XX
Adverse Events/Hosp.	XX	XX	XX	XX	XX	XX	XX	XX	XX	XX	XX	XX	XX	XX	XX	XX	XX	XX	XX	XX	XX	XX	XX	XX

1. Informed Consent must be signed prior to starting any prohibited drug washout.
2. The KPS will be determined at the time of the first visit for that week and the ADL will be completed at the time of the second visit for that week.
3. Locator Questionnaire only should be completed at Week 24, or at the time of an earlier termination.
4. Week 24 assessments which cannot be completed during week 24 of Stage II because of scheduling problems may be completed during week 25 with approval of sponsor.

APPENDIX C

PATIENT CONSENT FORM
AUTHORIZATION FOR TREATMENT WITH DRUG UNDER CLINICAL INVESTIGATION

PARTICIPANT'S NAME: ______________________ Date: ____/____/____

Study No.: _______ Patient Initials: _______

TITLE OF RESEARCH: A multi-center, double-blind, randomized, placebo-controlled study of the efficacy and safety of Ampligen® 400 mg IV twice weekly versus placebo in patients with severely debilitating chronic fatigue syndrome (CFS)/myalgic encephalomyelitis (ME)

PROTOCOL NO.: AMP-516

1. Introduction: You have been asked to voluntarily participate in this Ampligen® clinical research study as one of approximately 230 patients with CFS/ME.

   The investigational drug Ampligen® (poly I: poly $C_{12}U$) is a mismatched, double-stranded ribonucleic acid (dsRNA) which has been shown to modulate immune and antiviral activities.

2. Purpose: The purpose of this research study is to compare the efficacy and safety of the experimental/investigational drug Ampligen® versus placebo (normal saline) in the treatment of CFS/ME. You understand that data in humans indicate that Ampligen® may be effective in treating your condition.

3. Duration of Participation and Procedures: You understand that you will be in the study with two (2) distinct STAGES for a maximum of 64 weeks provided that you do not experience a dose limiting toxicity before the expiration of that period and that you comply with all requirements of Protocol AMP-516. You understand that your participation in this study does not give you any right to continue to receive Ampligen® after the conclusion of your treatment under this 64 week study. You understand that you must return to this clinic two (2) days each week while participating in the study.

You understand that missing more than two (2) consecutive doses or 10% or more of your total doses while on study is discouraged and may lead to the discontinuation of drug therapy. You understand that while receiving infusions, fewer than two (2) days or greater than a four (4) day interval between doses is discouraged.

You understand that if you are a female of child-bearing potential, you must not be nursing and a negative pregnancy test is required within two (2) weeks prior to receiving study drug and that you must practice effective birth control (birth control pills, intrauterine device, or diaphragm) beginning 1 month prior to the time of your pregnancy test, continue through baseline, continue throughout the period you are receiving study medication and until four (4) weeks after the last infusion. You understand that you will undergo a pregnancy test every eight (8) weeks throughout the study. You understand that animal studies indicate there may possibly be harm to unborn animals when their mothers were treated with Ampligen®. You understand that it is not known whether similar harmful effects will occur in humans. You understand that if you become pregnant you must notify the investigator, Dr.____________________, immediately. You understand that if you become pregnant, you will be removed from the study.

You understand that you will have a physical examination, medical history, chest X-ray, two (2) activity monitor tests, two (2) or three (3) treadmill exercise tolerance tests with EKGs, psychometric and quality of life assessments, urine test, pregnancy test (if you are a female of child-bearing potential), and blood tests performed prior to starting the study.

You will also be asked to complete a form which will be used to try to contact you every six months for two years after the treatment phase of the study has been completed. This form will request information concerning your address and phone number and the addresses and phone numbers of friends and family members who would likely know your whereabouts if you moved. Long term follow-up of this study is necessary to determine if the drug treatments you receive have benefit after the treatment stops. This follow-up involves completion of the same six questionnaires that you complete during the treatment phase of the study. If you withdraw from the study early before completion of the end of STAGE II, you will also be asked to complete these same six questionnaires at approximately the date corresponding to Week 20, Week 40 and Week 64. In either case you will be asked to mail the completed questionnaires to Hemispherx Biopharma in a pre-stamped and addressed envelope.

If you are accepted into the study, you understand that you will enter STAGE I of this two (2) STAGE study as outlined below:

STAGE I: Duration = 40 weeks; Treatment = Ampligen® or placebo, Double-blind

1. Ampligen® 200 mg IV twice weekly for four (4) doses (Weeks 1 and 2) then 400 mg IV twice weekly for 76 doses (Weeks 3 to 40) or
2. Placebo IV twice weekly for 80 doses (Weeks 1 through 40).

During STAGE I, neither you nor your physician will know whether drug or placebo has been assigned to you. The assignment will be done by chance (like flipping a coin), and you will have an equal chance of being on Ampligen® or placebo. You understand that you are not to take any other medication , including over-the-counter drugs, without informing your physician and obtaining his/her approval.

STAGE II: Duration = 24 weeks; Treatment = Ampligen®

Patients who comply with the protocol requirements of STAGE I, regardless as to whether they received Ampligen® or placebo, are eligible to participate in a 24 week open label study. Patients will receive Ampligen® 200 mg IV twice weekly for four (4) doses (Weeks 1 and 2) then 400 mg IV twice weekly for 44 doses (Weeks 3 thru 24). This study does not provide for Ampligen® therapy beyond STAGE II (Week 24).

You understand that you must inform your physician of all medications which you are taking. You understand that drug interactions between Ampligen® and other drugs have not been evaluated; you are therefore at risk if you take medications without your physician's knowledge. If you are found to be taking prohibited drugs your physician may remove you from the study.

You understand that: 1) quality of life and healthcare utilization assessments will done at least every four (4) weeks, 2) a complete physical examination, a pregnancy test (if you are a female of child-bearing potential), blood tests, urine tests and psychometric tests will be conducted at least every eight (8) weeks and one additional blood test will be done at week 4 of STAGE I and STAGE II, 3) the activity monitoring test, treadmill exercise test and EKG will be done once during weeks 18-22 and twice during weeks 34-39 of STAGE I and once during weeks 20 to 23 of STAGE II, 4) a chest X-ray will be performed at Week 40. During each activity monitoring test you will be asked to wear an activity monitor for 15 days. Wearing an activity monitor is similar to wearing a

waist wallet or beeper on a belt. The monitor's size is approximately 2 x 2 x 0.75 inches, which is smaller than a pack of cigarettes. Treadmill exercise testing will be done on day 8 of the activity monitor testing cycles. You understand that a physical examination will be performed every eight (8) weeks while on study. You understand that the average blood test will require that approximately 50 ml (equivalent to 10 teaspoonfuls) of blood will be drawn from you. A total of approximately 600 ml will be drawn over the course of the study.

If you are selected for the Immune panel (optional), an extra 25 ml (equivalent to 5 teaspoonfuls) of blood will be drawn at the beginning and end of STAGE I and at the end of STAGE II.

If your disease becomes worse during the study, you understand that you will be informed and your physician may decide to stop the treatment.

4. <u>Risks and Precautions</u>: You understand that you may experience the following risks and discomforts by participating in this study:

a. Your symptoms may: 1) not be relieved, 2) possibly become worse or 3) not respond at all to Ampligen®.

b. Ampligen® has been studied in several different types of animals including the rabbit, rat, dog and monkey. In the dog study, a small localized area of inflammation of the outer lining of the heart and destruction at the end of a rib were associated with Ampligen® treatment. These findings were not seen in studies evaluating rabbits, rats and monkeys. The meaning of these findings in the dog and how they relate to human clinical experience is not presently known. Liver damage and problems in making blood cells were observed in studies performed in dogs, rats and monkeys. Increases in thyroid activity were also seen in a study done in monkeys. In other studies of Ampligen® a decrease was seen in the number of unborn baby rabbits. Also, the weights of unborn baby rabbits and rats was less. Ampligen® treatment did not cause birth defects in the unborn baby rats and rabbits.

c. The side effects which have been noted in past studies with Ampligen® include tachycardia (rapid heart rate), arrhythmias (irregular heart beats), hepatic toxicity (liver damage), anaphylactoid reactions (flushing, rash, itching, low blood pressure and shortness of breath), fever, nausea, headache, joint or muscle pain, and fatigue. Other side effects may include decrease in the number of blood platelets (cells responsible for blood clotting) and white blood cells (cells responsible for fighting infection), dizziness, confusion, elevation of

kidney function tests which may indicate damage to the kidney, and occasional temporary hair loss. A severe local reaction with marked swelling and severe pain at the infusion site was seen in one patient following leakage of the Ampligen® solution into the surrounding tissue. A patient experienced a thrombosis (blood clot) of the superior vena cava (vein that empties into the heart) in the location of the surgically inserted catheter.

d. As with all medications, there may be unanticipated side effects. If you have any side effects, you will immediately notify:

____
Investigator

(______) ______________________
Phone Number

e. During the collection of blood samples, you understand that you may experience pain, bruising, bleeding, and/or infection at the site on your arm where blood is taken.

f. You understand that you will be notified of any significant new findings which may relate to your willingness to continue to participate in this study.

5. <u>Benefits</u>: You understand that a possible benefit to you by participating in this study is improvement of or relief from the symptoms of CFS/ME, but you also understand there is no guarantee that these benefits will happen.

You will receive very close monitoring and attention by the study staff during the months in which you are involved in this study.

Your reaction to the study medication may better facilitate the treatment of this disease in the future; this may benefit other patients as well as yourself.

6. <u>Alternative Treatment</u>: You understand that there is no approved therapy for the treatment of CFS/ME although certain specific symptoms may be treatable by other therapies.

7. <u>Confidentiality</u>: You hereby give your permission to your physician to release your medical records and information that pertains to this study to Hemispherx Biopharma, the Sponsor, representatives of the Sponsor and the Food and Drug Administration. You understand that medical records and information which reveal your identity will remain confidential, except that they will be provided as noted above or as they may be required by law.

8. Compensation: You have been advised that if you experience any physical injury due to these treatments or procedures, you should contact your Investigator, Dr.__________________________ at (_____)_____________, who is prepared to provide or obtain appropriate medical treatment. Neither the Investigator nor the Hospital nor Hemispherx Biopharma are aware of any compensation that is available for such physical injury. Neither the hospital nor the Investigator nor Hemispherx Biopharma will assume financial responsibility for medical treatment.

9. Voluntary Participation: Your participation in this study is voluntary. You are free to withdraw at any time without penalty or loss of benefits regarding your future care. You understand that your Investigator and/or the Sponsor have the right to withdraw you from the study, without regard to patient consent, at any time if required, for example, by a change in your medical condition, withdrawal of approval of this study, a change in the protocol duration, failure to comply with the dosing and testing requirements of the protocol, use of prohibited medications or other reasons.

You understand that any questions regarding this study may be addressed to your Investigator, Dr. ______________________ at (_____)_____________. If you feel there is any violation of your rights, you may call the Chairman of the Institutional Review Board, ________________________ at (_____)_____________.

10. Witnessing and Signatures: You have read this consent form before signing it. You have been offered ample opportunity to ask questions and have received answers that are to your full satisfaction. You voluntarily consent to participate in this study. You hereby agree to permit your Investigator, Dr.________________________ and such Associates and Assistants as he/she may designate to perform upon you (or upon the above named participant) the investigational treatment described in this agreement. You will receive a copy of this form when you sign it.

I understand that all responsibilities which accrue to either me or the Sponsor during the course of this study are fully disclosed and contained within this document and I have made my decision to participate based solely upon the information in this form.

______________________________ ______________
Patient's Signature Date

______________________________ ______________
Witness's Signature Date

______________________________ ______________
Investigator or his/her Designee Date

APPENDIX D

Case Definition of CFS

This definition is intended to serve as the basis for epidemiologic and clinical studies of the chronic fatigue syndrome. Although it may be a useful guide for the evaluation of a patient with a suggestive illness, the definition remains sufficiently nonspecific that it cannot confirm or deny the diagnosis of chronic fatigue syndrome in an individual patient. Chronic fatigue syndrome remains a diagnosis of exclusion, and physicians must continue to maintain a high level of suspicion throughout the course of the illness that other, more occult conditions may be causing the symptoms. A case of CFS must fulfill:

Major criteria 1 and 2, and the following minor criteria-- $\geq$ 6 of the 11 symptom criteria PLUS $\geq$ 2 of the 3 physical criteria;

OR

$\geq$ 8 of the 11 symptom criteria.

MAJOR CRITERIA

1. Acute or subacute onset of persistent or relapsing, debilitating fatigue or easy fatiguability in a person who has no previous history of similar symptoms, that does not improve with rest and that is severe enough to reduce or impair average daily activity below 50% of the patient's premorbid activity level for a period of at least 6 months.

2. Reasonable exclusion of other clinical conditions that may produce similar symptoms, based upon history, physical examination, and appropriate laboratory findings: 1) malignancy; 2) autoimmune disease; 3) localized infections (such as occult abscess), 4) chronic or subacute bacterial diseases (such as endocarditis, Lyme disease, or tuberculosis), fungal diseases (such as histoplasmosis, blastomycosis, or coccidiomycosis), and parasitic diseases (such as toxoplasmosis, amebiasis, giardiasis, or helminthic infestations); 5) AIDS or AIDS-related complex (ARC); 6) chronic inflammatory diseases (such as sarcoidosis, Wegener's granulomatosis or chronic hepatitis); 7) neuromuscular diseases (such as multiple sclerosis or myasthenia gravis); 8) endocrine diseases (such as hypothyroidism, Addison's disease, Cushing's syndrome, or diabetes mellitus); 9) side effects of chronic medications or other toxic agents (such as chemical solvents or heavy metals); 10) other chronic pulmonary, cardiac, gastrointestinal, hepatic, renal, hematologic,

neurological, musculoskeletal diseases; or 11) a major depressive disorder with psychotic or melancholic features; bipolar affective disorders; schizophrenia of any subtype; delusional disorders of any subtype; dementias of any subtype; anorexia nervosa; or bulimia nervosa. A recommended minimum laboratory evaluation for other possible causes should include the following: 1) complete blood count, differential, and platelet count; 2) erythrocyte sedimentation rate; 3) antinuclear antibody; 4) serum electrolytes; 5) glucose; 6) creatinine, blood urea nitrogen; 7) calcium, phosphorous; 8) total bilirubin, alkaline phosphatase, SGOT (AST), SGPT (ALT); 9) thyroid stimulating hormone level; 10) urinalysis; and 11) intermediate strength PPD.

MINOR CRITERIA

A. Symptom criteria

To fulfill a symptom criterion, a symptom must have begun at or after the time of onset of increased fatiguability, and must have persisted or recurred over a period of at least 6 months (the individual symptoms may or may not have occurred simultaneously):

1. Mild fever (oral temperature between 99.5 degrees F and 101.5 degrees F, if measured) and/or chills. Note: oral temperature of >101.5 degrees F is not commonly associated with CFS and should prompt studies for other causes).

2. Sore throat.

3. Painful anterior or posterior cervical or axillary lymph nodes.

4. Unexplained generalized muscle weakness.

5. Prolonged (≥ 24 hours) generalized fatigue following levels of exercise that would have been easily tolerated in the patient's premorbid state.

6. Headaches (of a type, severity, or pattern that is different from headaches suffered in the patients premorbid state).

7. Muscle discomfort/myalgias.

8. Migratory arthralgias without joint swelling or redness.

9. Neuropsychological complaints (forgetfulness, excessive irritability, confusion, dizziness, difficulty thinking, inability to concentrate, depression).

10. Sleep disturbance (hypersomnia, insomnia, difficulty falling asleep, early morning awakening).

11. Description of the main symptom complex as initially developing over a few hours to a few days.

B. Physical Criteria

Documented in a physician's office on at least two occasions, at least one month apart:

1. Low-grade fever (oral temperature between 99.7 degrees F and 101.5 degrees F, or rectal temperature 100.0 degrees F and 101.8 degrees F).

2. Non-exudative pharyngitis.

3. Palpable and/or tender anterior or posterior cervical or axillary lymphadenopathy (Note: lymph nodes > 2 cm in diameter suggest other etiologies. Further evaluation is warranted.)

APPENDIX E

PROCEDURE FOR DETERMINING KARNOFSKY PERFORMANCE SCORE (KPS)

I. <u>General Procedures for Determining Karnofsky Performance Scores</u>

The KPS is a global evaluation of the patient's ability to conduct daily activities, including work activities and self care activities. The KPS is sensitive to effective therapeutic intervention in chronic disease states. A KPS is assigned once every four (4) weeks, based upon a questionnaire (attached) completed by the patient and an interview which includes discussion of specific signs and symptoms, basic functional accomplishments (e.g., daily care activities), changes in activities and changes in medications taken. In addition, the assignment of the score may include discussions with a significant other (i.e., spouse, companion or custodian needed to care for patient's daily needs). The KPS score will be assigned by the investigator or in (his/her) absence by only one additional qualified designated individual at each site.

Karnofsky Performance Scale

100	Normal activity; no complaints; no evidence of disease
90	Able to carry on normal activity; minor signs or symptoms of disease
80	Normal activity with effort; some signs or symptoms of disease
70	Cares for self, unable to carry on normal activity or do active work
60	Requires occasional assistance but is able to care for most of needs
50	Requires considerable assistance for daily care
40	Disabled; unable to care for self, requires special care and assistance
30	Severely disabled; bedridden although death is not imminent
20	Very sick; hospitalization and/or nursing care is necessary; active supportive treatment is necessary
10	Moribund; fatal processes progressing rapidly
0	Dead

II. Recommendations on How to Perform a KPS Determination

A. The KPS determination will be done every four (4) weeks by the investigator or in his/her absence, the qualified designated other individual at each site. Note: KPS and Activity of Daily Living (ADL) evaluations should be done on separate visits.

B. The patient must first complete the KPS questionnaire and symptoms.

C. The questionnaire should be reviewed and the patient should be asked about any items needing clarification, including questions concerning the patient's self care or changes in activity, including how much time is spent in bed or in a reclining position and how much time is spent out of the house, doing household chores and reading, watching television, or talking on the phone.

D. The physician should assign the KPS in consultation with other health care workers who may also have interviewed the patient (e.g., infusion nurse, study coordinator).

E. The KPS must be rounded in increments of ten (10).

III. Comparison of Initial KPS Determination With Subsequent KPS Determinations

A. The initial KPS determination establishes a baseline, usually on a patient with whom the physician is unfamiliar.

B. This baseline is used as a focal point for the next interview in which questions are asked related to the improvement or worsening of specific signs, symptoms, or capabilities of the individual patient. These questions are the basis for the assignment of the post-baseline KPS.

C. Each subsequent KPS determination can rely on the previous interview in order to determine change in the patient's ability to conduct their daily activities.

HEMISPHERX BIOPHARMA, INC.

Karnofsky Performance Score (KPS)
QUESTIONNAIRE

To be Completed by CFS/ME Patient

1. Did you have any symptoms of your illness during the last week?

☐ Yes ☐ No

2. If yes, did your symptoms limit your ability to carry on normal activity during the last week?

☐ Yes ☐ No If yes, list the three (3) most severely impaired activities:

a. ____________________

b. ____________________

c. ____________________

3. During the last week were you able to do the following activities without help from anyone?

Laundry	☐ Yes	☐ No
Vacuuming	☐ Yes	☐ No
Yard maintenance	☐ Yes	☐ No
Grocery shopping	☐ Yes	☐ No

4. During the last week were you able to care completely for yourself on a regular basis without any help?

☐ Yes ☐ No

If no, list up to three (3) activities that you needed assistance with and indicate how much assistance was required by circ the most appropriate answer.

O = occasional-approximately 1-2 times per week, C = considerable-daily or almost daily, or T = total-multiple times d

	Circle one		
a. ____________________	O	C	T
b. ____________________	O	C	T
c. ____________________	O	C	T

5. What was your activity level last week?

☐ bedridden almost all the time (90-100%)
☐ inactive greater than 50% of the time
☐ inactive less than 50% of the time

6. Did you require 24 hour nursing care last week?

☐ Yes ☐ No

Patient to initial when complet

Initials:________________

APPENDIX F

EXERCISE TOLERANCE TEST (ETT) PROCEDURE

The following is an exercise tolerance test (ETT) procedure for all sites participating in the double-blind, randomized, placebo-controlled study of Ampligen® in CFS/ME:

I. Patient exclusion

a. Previous documented evidence of myocardial infarction or recent significant change in the resting EKG suggesting infarction or other acute cardiac events.

b. Current symptoms of coronary insufficiency (i.e. - angina pectoris and/or ST segment depression on EKG).

c. Evidence of uncontrolled atrial or frequent or complex ventricular ectopy, or myocardial conduction defect which would increase the risk of syncope (for example, second degree or higher A-V block).

d. History of congestive heart failure, suspected or known dissecting aneurysm, recent systemic or pulmonary embolus, severe valvular heart disease, ventricular aneurysm, active or suspected myocarditis or pericarditis, thrombophlebitis or intracardiac thrombi, or acute infection.

e. Evidence of moderate or severe obstructive pulmonary disease.

f. Resting diastolic blood pressure > 115 mm Hg or resting systolic blood pressure > 200 mm Hg.

g. Uncontrolled metabolic disease (e.g., diabetes, thyrotoxicosis, or myxedema).

h. Concurrent use of any beta blockers and/or bronchodilators which cannot remain at a stable dosage level during the eight (8) week washout period and continuing during baseline and STAGES I and II.

II. Equipment Required

a. Treadmill that is programmable for speed (1 mph minimum with 0.5 mph increments up to a speed of ≥ 5 mph) and grade (0 to ≥ 20%, incline in steps of 3%)

b. Continuous EKG monitoring (12 lead) during exercise testing. A resting 12 lead EKG will be done prior to each ETT at each evaluation period (baseline, Week 20 ± 2 and termination of STAGES I and II).

c. The treadmill will be calibrated according to the manufacturer's procedures/specifications prior to performing the initial ETT procedure at each site. Repeat calibration procedures will be performed at the frequency recommended by the manufacture. Written records will be maintained at each site and made available for review.

d. Pulse oximeter.

e. Resuscitation cart (defibrillator, laryngoscope, etc.)

III. <u>Patient Acclimation/Instruction</u>

a. Patient Instructions prior to testing:

1. The patient should avoid food, tobacco, alcohol and caffeine at least three (3) hours prior to testing.

2. Oily products, such as lotion or vapor rub, should not be used on the torso.

3. Comfortable clothing such as a short sleeve t-shirt, sweat pants and sneakers should be worn.

4. Patients should be rested for the assessment, avoiding significant exertion or exercise 24 hours prior to testing.

b. The entire procedure should be explained in detail to the patient in a group or individual setting.

c. Prior to exercise testing, a resting 12 lead EKG will be performed and the patient should be shown how to walk on the treadmill and actually be permitted to walk for a short period (less than 1 minute). The intention is to permit the patient to feel comfortable with the treadmill noise and the procedure for walking. This will aid in reducing anxiety.

d. The patient should be instructed to use his/her hands only for balance on the treadmill not to maintain position. It should be suggested that the patient not grip the bar with more than two or three fingers.

e. The patient should be permitted to sit down on a chair positioned on the treadmill for at least ten (10) minutes prior to beginning the rest data collection. This will minimize activity and therefore anxiety when you are ready to start the test.

IV. Patient Test Procedure

a. Attach EKG leads and establish the presence of a normal sinus rhythm.

b. Place the pulse oximeter lead on the patients forehead and secure it with a headband.

c. Assist in patient preparation for exercise. Help patient to stand and remove the chair from the treadmill. Position mouthpiece and nose clip.

d. Measure the rate of perceived exertion via the Borg Scale (See Borg Testing Protocol).

e. Blood pressure should be taken every two (2) minutes during the exercise protocol.

V. Exercise Testing Protocol

a. Initiate exercise according to the following protocol:

EXERCISE SCHEDULE

STAGE	MIN. OF EXERCISE (ELAPSED)	ELEVATION %	INCREMENT%	BELT SPEED (MPH)	INCREMENT (MPH)
I	0	0	-	2	-
II	2	3%	3%	2	0
III	4	6%	3%	2	0
IV	6	9%	3%	2	0
V	8	12%	3%	2	0
VI	10	15%	3%	2	0
VII	12	18%	3%	2	0
VIII	14	21%	3%	2	0
IX	16	21%	0	3	1
X	18	21%	0	4	1
XI	20	21%	0	5	1
XII	22	21%	0	5	0

Exercise may be extended after completing STAGE XII by maintaining the elevation at 21% and treadmill speed at 5 mph.

Blood pressure is to be measured during the last minute of each exercise stage.

VI. Borg Scale Testing Protocol

Category Rate of Perceived Exertion (RPE) Scale

6	
7	Very, very light
8	
9	Very light
10	
11	Fairly light
12	
13	Somewhat hard
14	
15	Hard
16	
17	Very Hard
18	
19	Very, very hard
20	

a. RPE scores will be taken at the end of each minute of exercise.

b. Patient instructions are as follows:

1) During the graded exercise, patients are to pay close attention to their exertion effort using the Borg Scale Testing Protocol.
2) This feeling should include the total amount of exertion and fatigue, combining all sensations and feelings of physical stress, effort and fatigue.
3) Patients should not be concerned with any one factor such as leg pain, shortness of breath or

exercise intensity, but should focus on the total, inner feeling of exertion.

4) Patients should not underestimate or overestimate, but try to be as accurate as possible.

VII. Pulse Oximetry

a. Allows for the continuous monitoring of arterial hemoglobin oxygen saturation (SAO_2). The finding of arterial oxygen desaturation indicates mismatching of ventilation/perfusion.

VIII. Indications for Test Termination by Operator

a. Progressive angina
b. Ventricular tachycardia, atrial fibrillation or flutter
c. Any significant drop (20 mm Hg) of systolic blood pressure or a failure of the systolic blood pressure to rise with an increase in exercise load
d. Lightheadedness, confusion, ataxia, pallor, cyanosis, nausea, or signs of severe peripheral circulatory insufficiency
e. > 4 mm horizontal or downsloping ST depression or elevation (in the absence of other indicators of ischemia)
f. Onset of second or third degree A-V block
g. Increasing ventricular ectopy, multiform PVCs, or R on T PVCs
h. Excessive rise in blood pressure: systolic pressure > 250 mm Hg; diastolic pressure > 120 mm Hg
i. Chronotropic impairment
j. Sustained supraventricular tachycardia
k. Exercise-induced left bundle branch block
l. Physical signs/symptoms of patient exhaustion and subject requests to stop
m. Failure of the monitoring system
n. SAO_2 declines below 87%

APPENDIX G

INVESTIGATOR ASSESSMENT OF CFS SIGNS AND SYMPTOMS

DURING THE LAST 7 DAYS

		Absent	If Present check Severity		
			Mild	Moderate	Severe
1.	Sore throat	____	____	____	____
2.	Painful cervical or axillary lymph nodes	____	____	____	____
3.	Unexplained generalized muscle weakness	____	____	____	____
4.	Muscle discomforts or aches	____	____	____	____
5.	Prolonged $\geq$ 24 hrs.) generalized fatigue	____	____	____	____
6.	Generalized headaches	____	____	____	____
7.	Migratory painful joints without swelling or redness	____	____	____	____
8.	Nonexudative inflammation of pharynx	____	____	____	____
9.	Palpable or tender cervical or axillary lymph nodes	____	____	____	____
10.	Areas of lost or depressed vision	____	____	____	____
11.	Visual intolerance of light	____	____	____	____
12.	Forgetfulness	____	____	____	____
13.	Excessive Irritability	____	____	____	____
14.	Confusion	____	____	____	____
15.	Difficulty thinking	____	____	____	____
16.	Inability to concentrate	____	____	____	____
17.	Depression	____	____	____	____
18.	Excessive sleep	____	____	____	____
19.	Inability to sleep	____	____	____	____
20.	Mild fever (Oral: 99.5°-101.5°F)	____	____	____	____

Date ____________________ Interviewer's Signature ________________________________

Appendix H

LOCATOR QUESTIONNAIRE

PROTOCOL NO.: AMP 516

INVESTIGATOR SITE NUMBER: ________

PATIENT NUMBER: ________

PATIENT INITIALS: ________

INTERVIEWER INITIALS: ________

INTERVIEW DATE: ___/___/___

STUDY WEEK NUMBER: ____________________ Check if STAGE I ☐ or STAGE II ☐

(If Baseline, write in Baseline)

INTERVIEWER READ: At the end of the treatment period we will be contacting you approximately every 6 months for 2 years as part of our effort to study the effectiveness of this program. You can help us now by giving us some information on how best to contact you later on. Your name and other information will be kept confidential in a locked file. It will not be given to anyone outside our research staff. We will not tell any contact anything except that you have been asked to take part in a "health study."

1. First, please tell me your full legal name.

   Name: ____
   First Middle Last (Maiden, if female)

2. Do you use any other names, nicknames, or aliases? ☐ Yes ☐ No

   IF "YES": Please tell me what these are.

   (1)____________________ (2) ____________________

   (3)____________________ (4) ____________________

(USE BACK OF PAGE IF SPACE IS NEEDED FOR ADDITIONAL NAMES)

3. What address would be best to use when we need to reach you again?

____
Street Address   Apt. #   P.O. Box

______________________________ ________ __________
City   State   Zip Code

(__________) __________________
Area Code   Telephone No.

3a. Whose address is this?

Name:____
First   Last   Maiden (if female)

Relationship:____

3b. Is this address a shelter? ☐ Yes ☐ No

3c. Is this the best mailing address? ☐ Yes ☐ No

IF "NO": Could you give me the best mailing address please?

____
Street Address   Apt. #   P.O. Box

______________________________ ________ __________
City   State   Zip Code

Whose address is this?

Name:____
First   Last   Maiden (if female)

Relationship:____

4. What phone number would be best to use to contact you?

(__________) __________________
Area Code   Telephone No.

Whose phone number is this?______________________________________
First   Last   Maiden (if female)

4a. If we were unable to reach you at that number, is there another good phone number to call:

☐ Yes ☐ No

IF "YES": What is that number please? (______) ______________________

Area Code Telephone No.

Whose phone number is this?______________________________

5. Can you tell me your mother's full name?

Name:____

First Maiden Last

Is she still living, would you give me her:

Address:____

Telephone No.: (______) ______________________

Area Code Telephone Number

6. Are there any other relatives who usually know how to reach you if you should move?

☐ Yes ☐ No

IF "YES", ASK: Please give me their:

(1) Name:____

Address:____

Telephone No.: (______) ______________________

Relationship:____

(2) Name:____

Address:____

Telephone No.: (______) ______________________

Relationship:____

7. Are there one or more friends who usually know how to reach you if you should move or leave our program: ☐ Yes ☐ No

IF "YES", ASK: Please give their names, addresses, and telephone numbers.

(1) Name:______________________________

Address:______________________________

Telephone No.: (_______) ______________________

Relationship:______________________________

(2) Name:______________________________

Address:______________________________

Telephone No.: (_______) ______________________

Relationship:______________________________

(3) Name:______________________________

Address:______________________________

Telephone No.: (_______) ______________________

Relationship:______________________________
