# Supplementary material for: A Double-Blind, Placebo-Controlled, Randomized, Clinical Trial of the TLR-3 Agonist Rintatolimod in Severe Cases of Chronic Fatigue Syndrome
Source: PLoS One. 2012 Mar 14;7(3):e31334. doi: 10.1371/journal.pone.0031334 (PMC3303772; doi:10.1371/journal.pone.0031334)
Supplement: Table S1 — Requirements for Entry into the Phase-III AMP-516 Clinical Study. (DOC) [file pone.0031334.s003.doc]

**Table S1. Requirements for Entry into the Phase-III AMP-516 Clinical Study**

| **A. Inclusion Criteria:** |
| --- |
| 1. A diagnosis of CFS, as defined by the Center for Disease Control (1988 CDC case definition) ≥12 months (Supplemental Table 2) |
| 2. Age range: ≥18 years old, ≤60 years old |
| 3. Males or non-pregnant, non-lactating females: Females must be of non-child-bearing potential (either post-menopausal for two (2) years or surgically sterile, including tubal ligation) or using an effective means of contraception (birth control pills, intrauterine device, diaphragm). Females who are less than two (2) years post-menopausal, those with tubal ligations, and those using contraception must have a negative serum pregnancy test within the two (2) weeks prior to the first study medication infusion. Females of child-bearing potential agree to use an effective means of contraception from four (4) weeks prior to the baseline pregnancy test until four (4) weeks after the last study medication infusion. |
| 4. A reduced quality of life as determined by a documented KPS (Supplemental Table 3) of 40 to 60 on three (3) occasions, each at least 14 days apart, during the twelve (12) weeks immediately preceding the start of study drug infusions. The KPS must be rounded in increments of ten (10). |
| 5. Ability to walk (minimum of 20 seconds) on the moving treadmill (grade=0%; belt speed=1 mph) on a minimum of two (2) occasions during the twelve (12) weeks immediately preceding study entry. |
| 6. Laboratory documentation (baseline or historical following onset of CFS) of a negative antinuclear antibody or a negative anti-ds DNA, a negative rheumatoid factor, and an erythrocyte sedimentation rate. |
| 7. Laboratory documentation that the patient is euthyroid (patients on thyroid replacement therapy must be on a stable dose during the eight (8) week washout period) based on thyroid profile (T_4_, T_3_, TSH, T_3_ uptake and Free T_4_ index) performed during baseline. |
| 8. Ability to provide written informed consent indicating awareness of the investigational nature of this study. |
| **B. Exclusion Criteria:** |
| 1. Inability to return to the investigator's site for scheduled infusions and evaluations during Stages 1 and 2 of the study. |
| 2. Chronic or intercurrent acute medical disorder or disease making implementation or interpretation of the protocol or results difficult or unsafe. |
| 3. Pregnant or lactating females |
| 4. Treatment with any of the following therapies within the eight (8) weeks immediately preceding the start of study baseline or during baseline: systemic glucocorticoids (ie, hydrocortisone, prednisone, etc.) or mineralocorticoids (ie, fludrocortisone [Florinef], etc.), interferons, interleukin-2, systemic antivirals, gamma globulin, or investigational drugs and experimental agents not yet approved for use in the United States. The patient was to give written consent prior to discontinuation of any drugs listed under this criterion. |
| 5. Prior participation in a study of Poly I:C_12_U. |
| 6. Medical necessity, as determined by the patient's private doctor or the principal investigator, to continue aspirin (ASA) or non-steroidal anti-inflammatory (NSAID) drugs for 20 consecutive days or for more than 10% of the study duration (i.e., 28 total days for Stage 1 and 17 total days for Stage 2). |
| 7. Ability to exercise over 18 minutes during any of the baseline ET procedures. |
| 8. Evidence or history of any exclusion criteria for the ET testing  a. Previous documented evidence of myocardial infarction or recent significant change in the resting electrocardiogram (ECG) suggesting infarction or other acute cardiac events.  b. Current symptoms of coronary insufficiency (i.e., angina pectoris and/or ST segment depression on ECG).  c. Evidence of uncontrolled atrial or frequent or complex ventricular ectopy, or myocardial conduction defect which would increase the risk of syncope (for example, second degree or higher A-V block).  d. History of congestive heart failure, suspected or known dissecting aneurysm, recent systemic or pulmonary embolus, severe valvular heart disease, ventricular aneurysm, active or suspected myocarditis or pericarditis, thrombophlebitis or intracardiac thrombi, or acute infection.  e. Evidence of moderate or severe obstructive pulmonary disease.  f. Resting diastolic blood pressure >115 mm Hg or resting systolic blood pressure >200 mm Hg.  g. Uncontrolled metabolic disease (e.g., diabetes, thyrotoxicosis, or myxedema).  h. Concurrent use of any beta blockers and/or bronchodilators which cannot remain at a stable dosage level during the eight- (8-) week washout period and continuing during baseline and Stages 1 and 2. |
| 9. History of alcohol or other substance abuse within two (2) years before the onset of the chronic fatigue and/or at any time afterward. |
| 10. History of suicidal ideation or a suicide attempt within two (2) years of baseline. |
| 11. Any past or current diagnosis of a major depressive disorder with psychotic or melancholic features; bipolar affective disorders; schizophrenia of any subtype; delusional disorders of any subtype; dementias of any subtype; anorexia nervosa; or bulimia nervosa. |
