# Supplementary material for: A Double-Blind, Placebo-Controlled, Randomized, Clinical Trial of the TLR-3 Agonist Rintatolimod in Severe Cases of Chronic Fatigue Syndrome
Source: PLoS One. 2012 Mar 14;7(3):e31334. doi: 10.1371/journal.pone.0031334 (PMC3303772; doi:10.1371/journal.pone.0031334)
Supplement: Table S2 — Clinical Trial Sites, Principal Investigators, and IRB. (DOC) [file pone.0031334.s004.doc]

**Table S2. Clinical Trial Sites, Principal Investigators, and IRB**

| **Principal Investigators** | **Study Site** | **Institutional Review Board (IRB)** ^*^ |
| --- | --- | --- |
| Lucinda Bateman, M.D., Salt Lake City, UT 84102 | 75BS | (IRB#1) |
| Joseph R. Bellesorte, D.O., Glen Mills, PA 19342 | 37BS | (IRB#1) |
| Paul J. Cimoch, M.D., Fountain Valley, CA 92708 | 52CI | (IRB#1) |
| Robert H. Keller, M.D., Hollywood, FL 33021 | 53KH | (IRB#1) |
| Charles W. Lapp, M.D., Charlotte, NC 28210 | 38LC | (IRB#1) |
| Alex J. Mercandetti, M.D., San Diego, CA 92103 | 54MS | (IRB #1) |
| Joseph John, Jr., M.D., New Brunswick, NJ 08903 | 57JN | (IRB#2) |
| Morris Papernik, M.D., Chicago, IL 60612 | 58PC | (IRB#1) |
| Daniel L. Peterson, M.D., Incline Village, NV 89452 | 13PI | (IRB#1) |
| Richard N. Podell, M.D., Springfield, NJ 07081 | 56PN | (IRB#1) |
| Bruce E. Stein, M.D., Manchester, CT 06040 | 65SM | (IRB#1) |
| Leslie vH. Taylor, M.D., Middleton, WI 53562 | 64TM | (IRB #3) |

^*^ This study was conducted in accordance with the ethical principles of the Declaration of Helsinki with protocol review by the following IRBs: IRB #1: Essex Institutional Review Board, Inc., 121 Main Street, Lebanon, New Jersey 08833, IRB #2: The UMDNJ – RWJMS IRB, 97 Paterson Street New Brunswick, NJ 08903, #3: Dean Institutional Review Board, 2711 Allen Boulevard, Middleton, WI 53562.
