# Supplementary material for: A Double-Blind, Placebo-Controlled, Randomized, Clinical Trial of the TLR-3 Agonist Rintatolimod in Severe Cases of Chronic Fatigue Syndrome
Source: PLoS One. 2012 Mar 14;7(3):e31334. doi: 10.1371/journal.pone.0031334 (PMC3303772; doi:10.1371/journal.pone.0031334)
Supplement: Table S3 — Clinical Significance of Karnofsky Performance Scale Scores. (DOC) [file pone.0031334.s005.doc]

**Table S3. Clinical Significance of Karnofsky Performance Scale^1^ Scores**

| **Score Description** | |
| --- | --- |
| 100 | Normal activity; no complaints; no evidence of disease. |
| 90 | Able to carry on normal activity; minor signs or symptoms of disease. |
| 80 | Normal activity with effort; some signs or symptoms of disease. |
| 70 | Cares for self, unable to carry on normal activity or do active work. |
| 60 | Requires occasional assistance but is able to care for most of needs. |
| 50 | Requires considerable assistance for daily care. |
| 40 | Disabled; unable to care for self, requires special care and assistance. |
| 30 | Severely disabled; bedridden although death is not imminent. |
| 20 | Very sick; hospitalization and/or nursing care is necessary; active support treatment is necessary. |
| 10 | Moribund; fatal processes progressing rapidly. |
| 0 | Dead. |

^1^ KPS is a physician assessment of disability. Used as an assessment of group status, smaller shifts are expected than individual responses to disease intervention. A KPS of 40 to 60 was required for study admission.
