# Supplementary material for: A Double-Blind, Placebo-Controlled, Randomized, Clinical Trial of the TLR-3 Agonist Rintatolimod in Severe Cases of Chronic Fatigue Syndrome
Source: PLoS One. 2012 Mar 14;7(3):e31334. doi: 10.1371/journal.pone.0031334 (PMC3303772; doi:10.1371/journal.pone.0031334)
Supplement: Table S4 — Treadmill Exercise Testing Protocol. (DOC) [file pone.0031334.s006.doc]

**Table S4. Treadmill Exercise Testing Protocol**

| **Stage** | **Minutes of Exercise (Elapsed)** | **Elevation**  **%** | **Increment**  **%** | **Belt Speed**  **(MPH)** | **Increment**  **(MPH)** |
| --- | --- | --- | --- | --- | --- |
| I | 0 | 0 | - | 2 | - |
| II | 2 | 3 | 3 | 2 | 0 |
| III | 4 | 6 | 3 | 2 | 0 |
| IV | 6 | 9 | 3 | 2 | 0 |
| V | 8 | 12 | 3 | 2 | 0 |
| VI | 10 | 15 | 3 | 2 | 0 |
| VII | 12 | 18 | 3 | 2 | 0 |
| VIII | 14 | 21 | 3 | 2 | 0 |
| IX | 16 | 21 | 0 | 3 | 1 |
| X | 18 | 21 | 0 | 4 | 1 |
| XI | 20 | 21 | 0 | 5 | 1 |
| XII | 22 | 21 | 0 | 5 | 0 |
