# Supplementary material for: A Double-Blind, Placebo-Controlled, Randomized, Clinical Trial of the TLR-3 Agonist Rintatolimod in Severe Cases of Chronic Fatigue Syndrome
Source: PLoS One. 2012 Mar 14;7(3):e31334. doi: 10.1371/journal.pone.0031334 (PMC3303772; doi:10.1371/journal.pone.0031334)
Supplement: Table S5 — Demographic Characteristics of the Rintatolimod (Poly I:C12U) versus Placebo Cohorts. (DOC) [file pone.0031334.s007.doc]

**Table S5. Demographic Characteristics of the Rintatolimod (Poly I:C_12_U) versus Placebo Cohorts**

| Parameter | Rintatolimod (n=117) | Placebo (n=117) | p-value |
| --- | --- | --- | --- |
| 1. Age, mean (years) | 43.4 + 9.20 | 43.5 + 10.1 | 0.925^1^ |
| 1. Age: Onset of CFS, mean (years) | 34.5 + 9.32 | 34.6 + 9.08 | 0.957^1^ |
| 1. Duration of CFS Symptoms, mean (years) | 9.6 + 5.36 | 9.7 + 6.08 | 0.825^1^ |
| 1. Time from CFS Diagnosis, mean (years) | 5.9 + 3.56 | 5.9 + 3.66 | 0.962^1^ |
| 1. Gender: % Female | 67.5 | 77.8 | 0.106^2^ |
| 1. Ethnicity: % Caucasian | 93.2 | 91.5 | 0.807^2^ |
| 1. Body Weight, mean (pounds) | 167+37.7 | 166+41.9 | 0.868^1^ |

^1^ 1-Factor (Treatment) ANOVA

^2^ 2-Tailed Fisher’s Exact Test
