# Supplementary material for: A Double-Blind, Placebo-Controlled, Randomized, Clinical Trial of the TLR-3 Agonist Rintatolimod in Severe Cases of Chronic Fatigue Syndrome
Source: PLoS One. 2012 Mar 14;7(3):e31334. doi: 10.1371/journal.pone.0031334 (PMC3303772; doi:10.1371/journal.pone.0031334)
Supplement: Table S6 — All Adverse Events Experienced by at Least 5% of Subjects in Either Treatment Group during the First 40 Weeks (Safety Population). (DOC) [file pone.0031334.s008.doc]

**Table S6.** All Adverse Events Experienced by at Least 5% of Subjects in Either Treatment Group during the First 40 Weeks (Safety Population)

|  | **Number (%) of subjects^1^** | | | | | |
| --- | --- | --- | --- | --- | --- | --- |
| **Preferred adverse event terms by body system** | **Rintatolimod**  **(N=117)** | | **Placebo**  **(N=117)** | | **Total**  **(N=234)** | |
| Body as a whole | 111 | (95) | 109 | (93) | 220 | (94) |
| Flu syndrome^2^ | 62 | (53) | 46 | (39) | 108 | (46) |
| Headache | 56 | (48) | 42 | (36) | 98 | (42) |
| Pain | 51 | (44) | 42 | (36) | 93 | (40) |
| Asthenia | 48 | (41) | 45 | (38) | 93 | (40) |
| Injection site reaction | 44 | (38) | 36 | (31) | 80 | (34) |
| Infection | 28 | (24) | 27 | (23) | 55 | (24) |
| Chest pain | 24 | (21) | 24 | (21) | 48 | (21) |
| Abdominal pain | 25 | (21) | 20 | (17) | 45 | (19) |
| Back pain | 23 | (20) | 17 | (15) | 40 | (17) |
| Fever | 22 | (19) | 16 | (14) | 38 | (16) |
| Chills^2^ | 26 | (22) | 11 | (9) | 37 | (16) |
| Accidental injury | 19 | (16) | 18 | (15) | 37 | (16) |
| Allergic reaction | 10 | (9) | 12 | (10) | 22 | (9) |
| Neck pain | 11 | (9) | 10 | (9) | 21 | (9) |
| Injection site pain | 7 | (6) | 7 | (6) | 14 | (6) |
| Face edema | 6 | (5) | 3 | (3) | 9 | (4) |
| Cardiovascular system | 53 | (45) | 44 | (38) | 97 | (41) |
| Migraine | 15 | (13) | 22 | (19) | 37 | (16) |
| Vasodilatation^2^ | 25 | (21) | 11 | (9) | 36 | (15) |
| Palpitation | 11 | (9) | 5 | (4) | 16 | (7) |
| Syncope | 9 | (8) | 3 | (3) | 12 | (5) |
| Digestive system | 71 | (61) | 72 | (62) | 143 | (61) |
| Nausea | 46 | (39) | 43 | (37) | 89 | (38) |
| Diarrhea | 25 | (21) | 19 | (16) | 44 | (19) |
| Dyspepsia | 12 | (10) | 20 | (17) | 32 | (14) |
| Vomiting | 12 | (10) | 10 | (9) | 22 | (9) |
| Gastrointestinal disorder | 8 | (7) | 5 | (4) | 13 | (6) |
| Anorexia | 8 | (7) | 4 | (3) | 12 | (5) |
| Constipation | 4 | (3) | 6 | (5) | 10 | (4) |
| Hemic and lymphatic system | 17 | (15) | 19 | (16) | 36 | (15) |
| Ecchymosis | 10 | (9) | 11 | (9) | 21 | (9) |
| Lymphadenopathy | 7 | (6) | 8 | (7) | 15 | (6) |
| Metabolic and nutritional disorders | 21 | (18) | 24 | (21) | 45 | (19) |
| Peripheral edema | 11 | (9) | 6 | (5) | 17 | (7) |
| Edema | 6 | (5) | 8 | (7) | 14 | (6) |
| Musculo-skeletal system | 49 | (42) | 39 | (33) | 88 | (38) |
| Myalgia | 32 | (27) | 27 | (23) | 59 | (25) |
| Arthralgia | 18 | (15) | 16 | (14) | 34 | (15) |
| Nervous system | 78 | (67) | 71 | (61) | 149 | (64) |
| Dizziness | 34 | (29) | 28 | (24) | 62 | (26) |
| Insomnia | 21 | (18) | 19 | (16) | 40 | (17) |
| Paresthesia | 19 | (16) | 16 | (14) | 35 | (15) |
| Thinking abnormal | 19 | (16) | 12 | (10) | 31 | (13) |
| Depression | 14 | (12) | 13 | (11) | 27 | (12) |
| Anxiety | 13 | (11) | 6 | (5) | 19 | (8) |
| Hypertonia | 10 | (9) | 7 | (6) | 17 | (7) |
| Somnolence | 7 | (6) | 6 | (5) | 13 | (6) |
| Nervousness | 6 | (5) | 3 | (3) | 9 | (4) |
| Tremor | 6 | (5) | 3 | (3) | 9 | (4) |
| Vertigo | 6 | (5) | 2 | (2) | 8 | (3) |
| Respiratory system | 61 | (52) | 56 | (48) | 117 | (50) |
| Rhinitis | 29 | (25) | 17 | (15) | 46 | (20) |
| Pharyngitis | 26 | (22) | 20 | (17) | 46 | (20) |
| Sinusitis | 15 | (13) | 21 | (18) | 36 | (15) |
| Dyspnea^2^ | 14 | (12) | 4 | (3) | 18 | (8) |
| Cough increased | 7 | (6) | 9 | (8) | 16 | (7) |
| Bronchitis | 7 | (6) | 6 | (5) | 13 | (6) |
| Skin and appendages | 51 | (44) | 53 | (45) | 104 | (44) |
| Rash | 27 | (23) | 26 | (22) | 53 | (23) |
| Pruritus | 20 | (17) | 13 | (11) | 33 | (14) |
| Sweating | 9 | (8) | 8 | (7) | 17 | (7) |
| Urticaria | 9 | (8) | 7 | (6) | 16 | (7) |
| Alopecia | 3 | (3) | 9 | (8) | 12 | (5) |
| Special senses | 41 | (35) | 35 | (30) | 76 | (32) |
| Taste perversion | 14 | (12) | 13 | (11) | 27 | (12) |
| Abnormal vision | 8 | (7) | 4 | (3) | 12 | (5) |
| Ear pain | 6 | (5) | 6 | (5) | 12 | (5) |
| Eye pain | 7 | (6) | 3 | (3) | 10 | (4) |
| Ear disorder | 6 | (5) | 3 | (3) | 9 | (4) |
| Urogenital system | 38 | (32) | 31 | (26) | 69 | (29) |
| Vaginitis | 13 | (11) | 13 | (11) | 26 | (11) |
| Urinary tract infection | 6 | (5) | 7 | (6) | 13 | (6) |
| Cystitis | 3 | (3) | 6 | (5) | 9 | (4) |

^1^ Subjects who experienced multiple adverse events in the same category are counted only once in each category. Subjects who experienced adverse events in multiple categories are counted only once in each category.

^2^ There are statistically significant differences between treatment groups in the proportions of subjects experiencing this event using the Fisher's Exact test.
