# Supplementary material for: A Double-Blind, Placebo-Controlled, Randomized, Clinical Trial of the TLR-3 Agonist Rintatolimod in Severe Cases of Chronic Fatigue Syndrome
Source: PLoS One. 2012 Mar 14;7(3):e31334. doi: 10.1371/journal.pone.0031334 (PMC3303772; doi:10.1371/journal.pone.0031334)
Supplement: Table S7 — Chronological Listing of Stage I Serious Adverse Events (SAEs). (DOC) [file pone.0031334.s009.doc]

**Table S7. Chronological Listing of Stage I Serious Adverse Events (SAEs)**

| **Treatment** | **Sex M/F** | **Age yrs** | **Serious adverse event (SAE)** | **Time from start of treatment to SAE, days** | **Duration of event if resolved, days** | **Action taken with respect to study drug** | **Causality as assessed  by the investigator** |
| --- | --- | --- | --- | --- | --- | --- | --- |
| Rintatolimod | F | 54 | Cerebral aneurysm | 99 | 52 | Dose held | No |
| Rintatolimod | M | 55 | Depression | 214 | 69 | None | No |
| Rintatolimod | F | 59 | Anxiety | 245 | Not resolved | None | No |
| Rintatolimod | F | 59 | Suicidal thoughts | 245 | 3 | None | No |
| Rintatolimod | F | 59 | Upper respiratory infection | 254 | 3 | None | No |
| Rintatolimod | F | 39 | Headache | 84 | 0 | None | No |
| Rintatolimod | F | 28 | Suicide attempt | 121 | 3 | None | Possible |
| Rintatolimod | F | 49 | Abscess | 124 | 3 | Dose held | No |
| Rintatolimod | M | 38 | Abdominal pain | 131 | 1 | Dose held | No |
| Rintatolimod | F | 51 | Abdominal pain | 150 | 50 | None | No |
| Rintatolimod | F | 39 | Pulmonary embolism | 194 | Not resolved | Dose held | Remote |
| Rintatolimod | F | 35 | Uterine fibroids | 59 | 92 | None | No |
| Rintatolimod | F | 35 | Cerebrovascu-lar accident | 221 | 5 | None | No |
| Rintatolimod | F | 36 | Paresthesia | 239 | 2 | None | No |
| Rintatolimod | M | 39 | Abdominal pain, gastric distention, 2 occurrences | 9, 15 | 3, 24 | None, None | Remote, No |
| Placebo | F | 44 | Abdominal pain | 271 | 6 | None | No |
| Placebo | F | 42 | Difficulty breathing, chest tightness | 117 | Not resolved | Dose held | Probable |
| Placebo | F | 57 | Accidental injury | 242 | Not resolved | N/A | No |
| Placebo | F | 35 | Epilepsia partialis continua, Seizures | 43, 251 | 2, Not resolved | Dose held, Dose held | No, Possible |
| Placebo | F | 45 | Cholelithiasis | 68 | 1 | Dose held | Remote |
| Placebo | F | 49 | Anxiety | 150 | 2 | None | No |
| Placebo | F | 49 | Depression | 172 | Not resolved | None | No |
