# Supplementary material for: A Double-Blind, Placebo-Controlled, Randomized, Clinical Trial of the TLR-3 Agonist Rintatolimod in Severe Cases of Chronic Fatigue Syndrome
Source: PLoS One. 2012 Mar 14;7(3):e31334. doi: 10.1371/journal.pone.0031334 (PMC3303772; doi:10.1371/journal.pone.0031334)
Supplement: Table S8 — Comparison of Intra-Group Mean ET Improvement Seen With Rintatolimod (PolyI:C12U) vs. Approved Drugs for Non-CFS Severe Exertional Fatigue. (DOC) [file pone.0031334.s010.doc]

**Table S8. Comparison of Intra-Group Mean ET Improvement Seen With Rintatolimod (PolyI:C_12_**U) vs. Approved Drugs for Non-CFS Severe Exertional Fatigue

| **Chronic Disease Indication** | **Drug (Placebo-Controlled Clinical Trial)** | **Group Analyzed** | **% Improvement Over Placebo** |
| --- | --- | --- | --- |
| Chronic Fatigue Syndrome | Rintatolimod (AMP-516) | Completed 40 Weeks | 14.0 |
| Chronic Fatigue Syndrome | Rintatolimod (AMP-516) | Intent-to-Treat | 11.8 |
| Chronic Congestive Heart Failure | Fosinopril [16] | 20 mg dosage group | 6.7 |
| Chronic Congestive Heart Failure | Captopril [17] | 150 mg dosage group | 6.2 |
| Chronic Angina | Ranolazine [18] (MARISA) | 500/1,000 mg Pooled Data ^1^ | 6.5 |
| Chronic Angina | Ranolazine [19] (CARISA) | 750/1,000 mg Pooled Data ^1^ | 5.9 |
| Pulmonary Arterial Hypertension^2^ | Tracleer (Breathe – 1) | 125 mg BID dosage group | 10.6 |
| Pulmonary Arterial Hypertension^2^ | Remodulin (PO1:04) | 1.25-22.5 mg/kg/min | 8.0 |
| Pulmonary Arterial Hypertension^2^ | Remodulin (PO1:05) | 1.25-22.5 mg/kg/min | 4.1 |
| Pulmonary Arterial Hypertension^2^ | Remodulin (PO1:04-05) | Pooled Data ^1^ | 6.1 |

^1^ Pooled data means results from both study groups were pooled.

^2^ Data from the FDA summary basis of approvals (6 minute walk)
