# Supplementary material for: A Double-Blind, Placebo-Controlled, Randomized, Clinical Trial of the TLR-3 Agonist Rintatolimod in Severe Cases of Chronic Fatigue Syndrome
Source: PLoS One. 2012 Mar 14;7(3):e31334. doi: 10.1371/journal.pone.0031334 (PMC3303772; doi:10.1371/journal.pone.0031334)
Supplement: Table S9 — Concomitant Medications Used for CFS Symptoms. (DOC) [file pone.0031334.s011.doc]

**Table S9. Concomitant Medications Used for CFS Symptoms**

| **Drug Categories** | **Example** |
| --- | --- |
| Analeptics | Provigil (modafinil) |
| Analgesics | Darvon (propoxyphene) |
| Anticonvulsants | Neurontin (gabapentin) |
| Antidepressants | Prozac (fluoxetine) |
| Antihistamines | Atarax (hydroxyzine) |
| Anti-infectives | Zithromax (azithromycin) |
| Anti-inflammatories | Advil (ibuprofen) |
| Antipyretics | Tylenol (acetaminophen) |
| Hypnotics | Ambien (zolpidem) |
